# Identification and functional characterization of small non-coding RNAs in *Xanthomonas oryzae *pathovar *oryzae*

**DOI:** 10.1186/1471-2164-12-87

**Published:** 2011-01-30

**Authors:** Hong Liang, Ying-Tao Zhao, Jie-Qiong Zhang, Xiu-Jie Wang, Rong-Xiang Fang, Yan-Tao Jia

**Affiliations:** 1 State Key Laboratory of Plant Genomics, Institute of Microbiology, Chinese Academy of Sciences, Beijing 100101, PR China; 2 National Center for Plant Gene Research, Beijing 100101, PR China; 3 Graduate School of the Chinese Academy of Sciences, Beijing 100039, PR China; 4 State Key Laboratory of Plant Genomics, Institute of Genetics and Developmental Biology, Chinese Academy of Sciences, Beijing 100101, PR China

## Abstract

**Background:**

Small non-coding RNAs (sRNAs) are regarded as important regulators in prokaryotes and play essential roles in diverse cellular processes. *Xanthomonas oryzae *pathovar *oryzae *(*Xoo*) is an important plant pathogenic bacterium which causes serious bacterial blight of rice. However, little is known about the number, genomic distribution and biological functions of sRNAs in *Xoo*.

**Results:**

Here, we performed a systematic screen to identify sRNAs in the *Xoo *strain PXO99. A total of 850 putative non-coding RNA sequences originated from intergenic and gene antisense regions were identified by cloning, of which 63 were also identified as sRNA candidates by computational prediction, thus were considered as *Xoo *sRNA candidates. Northern blot hybridization confirmed the size and expression of 6 sRNA candidates and other 2 cloned small RNA sequences, which were then added to the sRNA candidate list. We further examined the expression profiles of the eight sRNAs in an *hfq *deletion mutant and found that two of them showed drastically decreased expression levels, and another exhibited an Hfq-dependent transcript processing pattern. Deletion mutants were obtained for seven of the Northern confirmed sRNAs, but none of them exhibited obvious phenotypes. Comparison of the proteomic differences between three of the ΔsRNA mutants and the wild-type strain by two-dimensional gel electrophoresis (2-DE) analysis showed that these sRNAs are involved in multiple physiological and biochemical processes.

**Conclusions:**

We experimentally verified eight sRNAs in a genome-wide screen and uncovered three Hfq-dependent sRNAs in *Xoo*. Proteomics analysis revealed *Xoo *sRNAs may take part in various metabolic processes. Taken together, this work represents the first comprehensive screen and functional analysis of sRNAs in rice pathogenic bacteria and facilitates future studies on sRNA-mediated regulatory networks in this important phytopathogen.

## Background

As an emerging class of gene expression modulators, small non-coding RNAs (sRNAs) have been detected in almost all kingdoms of life and are gaining increasing attention because of their important roles in various physiological processes. With the rapid progress of research on bacterial transcriptome, hundreds of sRNAs have been identified. Subsequent functional analyses have revealed that these sRNAs regulate various cellular processes, such as stress responses [1], quorum sensing [2], life cycle differentiation [3] and virulence [4-7]. Systematic screen of sRNAs have been performed in diverse bacteria, such as *Escherichia coli *[8-11], *Salmonella enterica *[12], *Pseudomonas aeruginosa *[13] and many other bacterial species distantly related to *E. coli *[14-18]. These studies reveal that sRNAs are widely encoded in bacterial genomes, the discovery pace of bacterial sRNAs has continued to accelerate and the functions of increasing sRNAs are being elucidated [19].

Bacterial sRNAs are usually 50-500 nucleotides (nt) in length. Besides binding with proteins to modulate their activities, the majority of sRNAs regulate their target genes by base pairing and function as diffusible molecules [20]. The base pairing sRNAs can be further classified into two subgroups: *trans*-encoded sRNAs and *cis*-encoded sRNAs. Of them, *trans*-encoded sRNAs have been well-studied during the last two decades. These sRNAs are transcribed from the genomic loci which are physically unlinked to their target genes. *Trans*-encoded sRNAs usually regulate the translation or stability of their target mRNAs through partial and discontinuous complementarities. The *trans*-encoded sRNAs resemble the eukaryotic microRNAs in their ability to modulate mRNA stability and translation [19,20]. In addition, most of the *trans*-encoded sRNAs require the bacterial Sm-like protein, Hfq, to perform their regulatory functions [21]. Hfq plays important roles in sRNAs-mediated regulation by affecting the stability of sRNAs and facilitating the base-pairing between sRNAs and their target mRNAs [22]. The *hfq *mutant exhibits various phenotypes in many bacterial species, including reduced growth rate, changed pathogenicity and altered tolerance to stress conditions [23-28]. Another subgroup of antisense sRNAs is the *cis*-encoded sRNAs which are transcribed from the opposite strand of their target genes and regulate their target genes through complete complementarities [29]. Although most of the identified *cis*-encoded sRNAs are encoded by phages, plasmids and transposons [30], recent studies revealed that bacterial chromosomes also generate a large number of *cis*-encoded sRNAs. Besides, RNA regulators such as riboswitches and CRISPR (clusters of regularly interspaced short palindromic repeats) RNAs also play regulatory roles and exist widely in bacteria [20].

*Xanthomonas oryzae *pathovar *oryzae *(*Xoo*) is a Gram-negative bacterium that belongs to the gamma subdivision of Proteobacteria and is the causal agent of the bacterial blight of rice. *Xoo *has long been used as a model organism in studying plant pathology. Currently, the complete genomic sequences of three *Xoo *strains are available [31-33], allowing for genome-scale analysis. During the past few years, a number of regulatory genes were identified in *Xoo*, especially those involved in virulence and host cell recognition, but very little is known about sRNAs and sRNA-mediated regulations in this bacterium. *Bona fide *small regulatory RNAs have not yet been described in *Xoo*, although some house-keeping sRNAs, regulatory RNAs such as riboswitches [34] and CRISPR RNAs [35] were reported. In the *Xanthomonas *genus, only four sRNAs from *Xanthomonas campestris pv.campestris *(*Xcc*) [36], the causal agent of black rot disease of crucifers, and a plasmid transferred anti-sense sRNA from *Xanthomonas campestris *pv. *vesicatoria *(*Xcv*) [37] were reported previously. Therefore, the presence of sRNAs in *Xoo *genome and their regulatory functions remain to be elucidated.

Here, we conducted a global screen for sRNAs in the *Xoo *strain PXO99 by experimental cloning coupled with computational prediction. This work aimed: 1) to understand the number, genomic distribution and subgroups of *Xoo *sRNAs; 2) to examine the expression of some sRNAs for future studies, and 3) to screen for putative target genes of sRNAs of interest by a proteomic assay. In total, we obtained 65 putative sRNA candidates within the *Xoo *genome. Among them, the expression of eight sRNAs was experimentally confirmed, and three of them were determined to be Hfq-dependent sRNAs. We successfully constructed seven sRNA-deleted mutants, and proteomic analysis performed on three of these mutants indicated that the corresponding sRNAs are likely to be involved in various physiological pathways. The results of this study will facilitate future investigations on sRNAs functions in this important phytopathogenic bacterium.

## Results and Discussion

### Identification of candidate sRNA genes in *Xoo*

**Figure 1:**
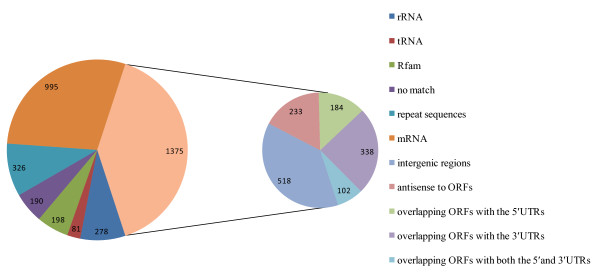
**Composition of the *Xoo *cDNA library**. The cDNA sequences were grouped according to their categories, and the number of clones in each category is shown. The uniquely mapped clones which have the potential to encode sRNA candidates were further classified based on the strands and their positions relative to the flanking ORFs.

A cDNA library of RNAs with the size ranging from 50 to 500 nt was generated from the *Xoo *strain PXO99 grown under standard laboratory conditions to study its population of sRNAs. As the expression of some bacterial sRNAs has been reported to be up-regulated around the stationary phase [8,11,38], we used total RNA extracted from the *Xoo *cells in the stationary phase (OD_600 _= 1.5) in this study. A total of 10,560 individual clones were pre-screened by hybridization on customer-made arrays to eliminate clones containing rRNA or tRNA sequences. Subsequently, 3,443 cDNA clones exhibiting low hybridization signals were sequenced. The obtained sequences were analyzed and classified according to their annotation and genomic locations (Additional file 1 and Figure 1). A total of 190 low-quality sequences were excluded from further studies either due to their short sequence lengths or lack of complete adapter sequences. There were still 278 and 81 cDNA clones corresponding to the fragments of rRNA and tRNA sequences, respectively, since the designed probes used in the array analysis did not cover these regions. By comparing with the seven types of riboswitch elements and four classes of house-keeping sRNAs of *Xoo *reported in the Rfam database [34], 198 cloned sequences representing two riboswitch elements and three house-keeping sRNAs were found, suggesting the validity of our library. Among these reported sRNAs, the stable transfer-messenger RNA (tmRNA) had the highest clone frequency (Table 1).

**Table 1:** Riboswitch elements and house-keeping small RNAs identified in our library

RNAs	number of clones in library
TPP	1
YybP-ykoY	2
6S	26
tmRNA	151
RNaseP_bact_a	18

Of the remaining 2,696 clones, 326 were mapped to multiple loci; thus their accurate genomic locations could not be determined without additional information. For the uniquely mapped clones, 995 of them were unambiguous fragments of mRNAs according to the open reading frame (ORF) annotations of the National Center for Biotechnology Information (NCBI). All these clones were then excluded from further investigation. The rest of the uniquely mapped 1,375 clones were categorized in detail. There were 518 (228 unique) clones derived from the IGRs and 233 (180 unique) cDNA clones were expressed from the opposite strand of ORFs. There were also 184 (153 unique) cDNA clones partially overlapped with the 5' untranslated regions (UTRs) and the coding sequences of ORFs. Since the majority of the transcription start sites of the protein coding genes were unknown in *Xoo*, these clones may represent partial mRNAs, riboswitches, or independent non-coding RNAs which may attenuate transcription or regulate translation initiation. In addition, 338 (219 unique) cDNA clones began within the ORFs and extended to the 3' UTRs of the ORFs; and 102 (83 unique) cDNA clones spanned the whole ORFs and overlapped with both the 5' and 3' UTRs. Detailed information on these sequences is listed in Additional file 2. We also used the Glimmer program [39] to screen whether any previously unannotated ORFs existed near or within these non-coding sequences. Sixty-eight putative ORFs were predicted by Glimmer and 28 predicted ORFs had canonical ribosome binding sites (RBS) [40]. A total of 23 (13 unique) cloned sequences overlapped with the predicted ORFs owing obvious RBS. The results are listed in Additional file 2. In brief, these results suggest that there are a large number of putative non-coding RNAs which can be classified into various categories in the *Xoo *genome although experimental evidence is necessary to confirm them.

In addition to the cloning approach, we also applied the bacterial sRNA prediction program SIPHT (sRNA identification protocol using high-throughput technologies) to computationally predict sRNAs in the *Xoo *PXO99^A ^genome [41]. A total of 269 sRNA candidates were predicted, of which 63 candidates were also identified by the cloning approach, thus were selected as sRNA candidates (Additional file 3). Both the direct cloning and the bioinformatic prediction approaches facilitated the detection of sRNAs in *Xoo*, providing us a large number of putative sRNA candidates for further studies. Recently, four novel sRNAs in the *Xcc *genome were reported [36], and three of them are conserved in the *Xoo *genome, of which, two sRNAs (sRNA-*Xcc*2, sRNA-*Xcc*4) were among our identified sRNA candidates, indicating that these two sRNAs may have important functions in *Xanthomonas*.

### Experimental verification and expression profiles of the sRNAs in *Xoo*

**Figure 2:**
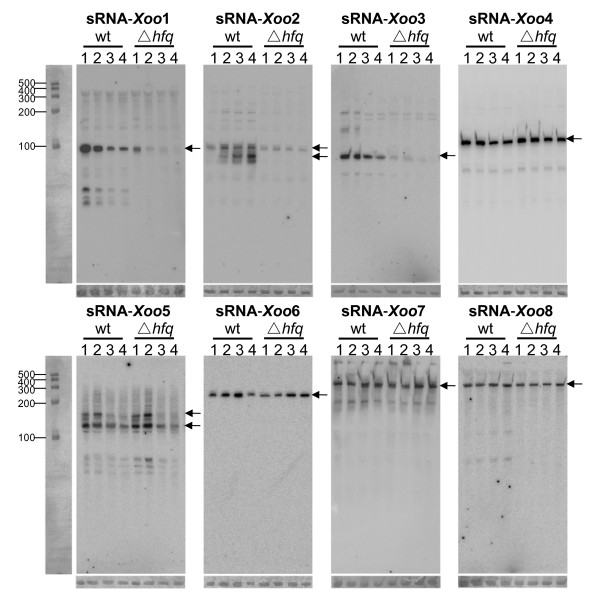
**Experimental verification and expression profiles of *Xoo *sRNAs**. *Xoo *sRNAs were verified and analyzed by Northern blotting. RNA samples were isolated from wild-type (wt) and Δ*hfq *mutant cells cultured in PSA medium at different growth phases. The OD_600 _values of the cultures are represented by numbers (1: OD_600 _= 0.5, 2: OD_600 _= 1.0, 3: OD_600 _= 1.5, 4: OD_600 _= 2.0). The DNA probes used in Northern analysis were complementary to the sRNA genes. Arrows indicate the predominant bands. Standard RNA markers are shown on the left. rRNAs served as control for RNA loading and RNA integrities are shown on the bottom panel.

Eight newly-identified sRNA candidates were selected for experimental confirmation. To investigate whether the bioinformatic prediction may miss real sRNAs, two cloned sequences (sRNA-*Xoo*2, sRNA-*Xoo*4) without prediction evidence were also selected for experimental confirmation. For these ten putative sRNA candidates, six of them were generated from the IGRs, and the other four candidates were from the UTR regions. Northern blot analyses were performed to verify these putative sRNA candidates using total RNA isolated from the wild-type strain at various growth phases. Eight sRNA candidates were repeatedly detected by Northern blot analysis and designated as sRNA-*Xoo*1, sRNA-*Xoo*2, sRNA-*Xoo*3, sRNA-*Xoo*4, sRNA-*Xoo*5, sRNA-*Xoo*6, sRNA-*Xoo*7 and sRNA-*Xoo*8, as showed in Figure 2. The sizes of the RNA molecules detected by Northern blotting were roughly in agreement with the length determined by the cloned or 5' RACE mapped sequences, which ranged from 78 nt to 365 nt (Table 2 and Additional file 4). The detailed information of these sRNAs is listed in Table 2.

**Table 2:** Summarized information on sRNAs verified in this study

sRNAs	strand	upstream gene	downstream gene	5' end	3' end	size (nt)	**No**.	prediction
sRNA-*Xoo*1	+	PXO_02602	PXO_02603	4451914^a^	4452016^b^	103^c^; ~100^d^	1	NC-211
sRNA-*Xoo*2	+	PXO_01687	PXO_01686	3205976^b^	3206053^b^	78 ^e ^~100^d^;	1	N
						~85^d^		
sRNA-*Xoo*3	+	PXO_03614	PXO_03613	250669^a^	250761^b^	93^c^; ~80^d^	2	NC-137
sRNA-*Xoo*4	-	PXO_05774	PXO_02847	4955974^a^	4955830^b^	145^c^; ~150^d^	99	N
sRNA-*Xoo*5	+	PXO_00354	PXO_00353	2045785^a^	2045908^b^	124^c^;~130^d^;	2	NC-123
						~100^d^		
sRNA-*Xoo*6	-	PXO_03506	PXO_03507	5217958^b^	5217771^b^	188 ^e ^~250^d^	7	NC-248
sRNA-*Xoo*7	-	PXO_04745	PXO_04746	1379160^a^	1378796^b^	365^c^;~300^d^	1	NC-40
sRNA-*Xoo*8	+	PXO_04362	PXO_04361	1067954^b^	1068276^b^	323^e^;~300^d^	6	NC-87

^a^Ends determined by 5' RACE mapping (5' RACE results are given in Additional file 4)

^b^Ends determined by cloned sequences.

^c^Size determined by RACE analysis

^d^Size observed in Northern blot analysis.

^e^Size determined by cloned sequences.

No.: number of cloned sequences in our cDNA library

The sRNA-*Xoo*2 and sRNA-*Xoo*7 are only conserved among *Xoo *strains and its closely related pathovar *X. oryzae *pv *oryzicola*, although their flanking genes are well conserved in other *Xanthomonas *species (Table 3). This results indicated that these two sRNAs may have originated recently and have species-specific functions. To the contrary, the other six sRNAs (sRNA-*Xoo*1, 3, 4, 5, 6, 8) are conserved among *Xanthomonas *(Table 3). To determine whether these eight sRNAs are newly identified, a BLAST search was performed against the Rfam database http://www.sanger.ac.uk/Software/Rfam/ and small RNA database http://biobases.ibch.poznan.pl/ncRNA/, none of these *Xoo *sRNA genes had similarities with any reported sRNAs. But it is possible that evolutionarily related sRNAs may also lack sequence similarity in different bacteria species. Then the conservation of their flanking genes was analyzed since sRNA and their flanking genes might be evolutionarily related but differed in the pace of diversity [19]. The blastx searches revealed that two flanking genes (*lpxC *and *ftsZ*) of sRNA-*Xoo*8 were highly conserved in many bacteria species although the intergenic region evolved rapidly. In *Pseudomonas aeruginosa *PAO1, a predicted sRNA annotated as PA4406.1 [42] was found between *lpxC *and *ftsZ*. These results indicated that sRNA-*Xoo*8 may be related to PA4406.1 evolutionarily. For the other seven *Xoo *sRNAs, evolutionarily related sRNAs have not been identified in other bacteria species, indicating that they are newly identified sRNAs in bacteria.

**Table 3:** Conservation of the identified sRNAs in closely related species

species	*Xooc*		*Xcc*		*Xoo*		*Xcv*	*Xac*		*Xf*
strain	BLS256	8004	ATCC33913	B100	KACC10331	MAFF311018	85-10	306	9a5c	Temecule1
sRNA-*Xoo*1	N	91%	91%	91%	100%	100%	95%	95%	N	N
sRNA-*Xoo*2	95%	N	N	N	100%	100%	N	N	N	N
sRNA-*Xoo*3	98%	94%	94%	94%	100%	100%	96%	95%	N	N
sRNA-*Xoo*4	95%	92%	92%	92%	100%	100%	93%	93%	N	N
sRNA-*Xoo*5	99%	94%	94%	94%	99%	100%	99%	98%	N	N
sRNA-*Xoo*6	96%	92%	92%	92%	100%	99%	94%	94%	N	N
sRNA-*Xoo*7	99%	N	N	N	100%	99%	N	N	N	N
sRNA-*Xoo*8	97%	84%	84%	84%	100%	100%	88%	89%	N	N

*Xooc: Xanthomonas oryzae *pv *oryzicola*; *Xcc*: *Xanthomonas campestris *pv. *campestris*; *Xoo*: *Xanthomonas oryzae *pv. *oryzae*; *Xcv*: *Xanthomonas campestris *pv. *vesicatoria*; *Xac*: *Xanthomonas axonopodis pv. citri; Xf: Xylella fastidiosa; *N: no similar sequence

**Figure 3:**
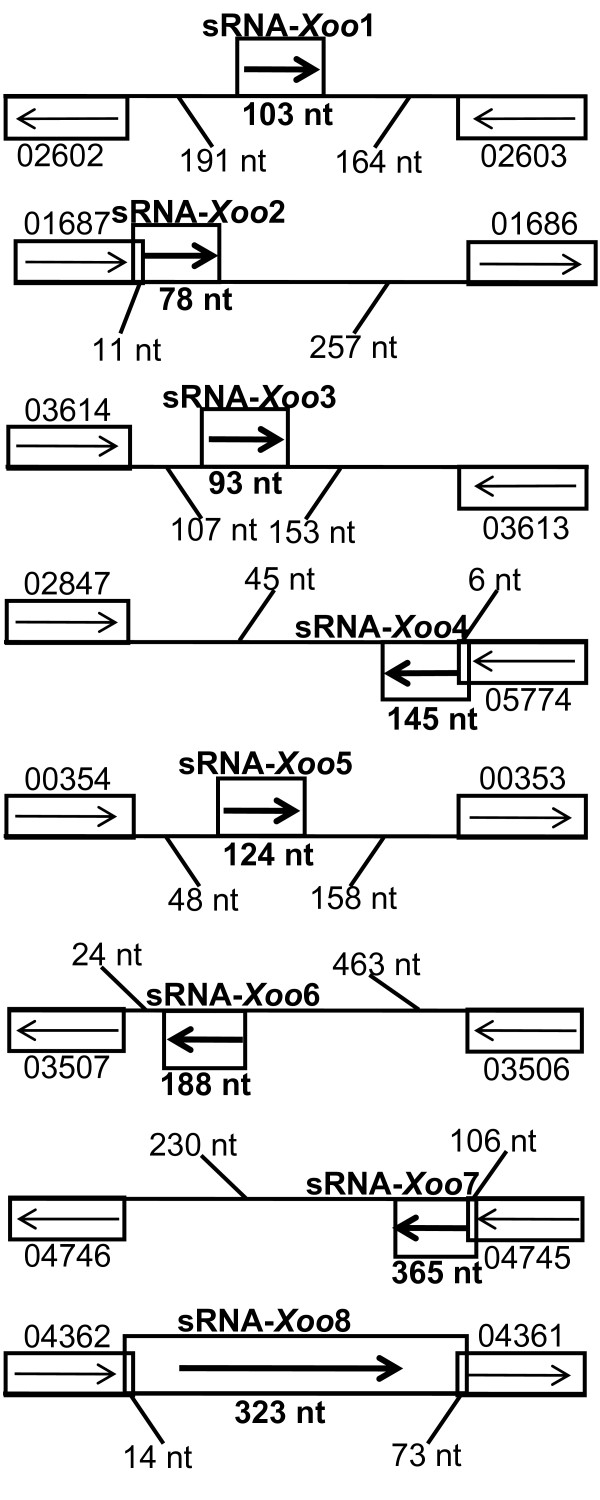
**Schematic representation of the genomic positions of the verified sRNA genes**. The genomic positions of the eight sRNA genes in the *Xoo *strain PXO99 are shown. The orientation of each sRNA gene and the flanking ORFs (the gene ID is listed) are indicated by the arrow. The lengths of the sRNA genes, the distances between the sRNA gene and its flanking genes are also indicated.

Among the eight experimentally verified sRNAs, two sRNAs (sRNA-*Xoo*2, sRNA-*Xoo*4) were overlooked in our bioinformatic search, probably due to their close proximity to the adjacent coding sequences, whereas the clone-based method enabled us to identify these two sRNAs successfully (Table 2). Among the eight sRNAs, half of them were encoded within the IGRs, while the other four sRNAs overlapped with adjacent genes (Figure 3). In addition, the other two sRNA candidates (PXO_03433-03434 and PXO_00355-00356) which were oriented in IGRs could not be detected by Northern blot analysis under the test conditions. These candidates either showed multiple faint bands without predominant bands or failed to be detected completely. We considered these two sRNA candidates to be likely transcribed at extremely low levels or not transcribed at all under the tested conditions.

The expression profiles of these identified sRNAs were also revealed by Northern blotting. Two sRNAs (sRNA-*Xoo*2, sRNA-*Xoo*5) showed size variations, indicating the presence of a precursor or processing event according to the growth phases. For sRNA-*Xoo*2, two transcripts were detected. The abundance and the relative ratio between these two transcripts varied with the growth phase, as the abundance of the longer transcript decreased when entering the stationary phase, whereas that of the shorter one increased at the same time. The shorter transcript may be a processed product or an active version of the longer one. For sRNA-*Xoo*5, two transcripts were detected in the early growth phases, while in the later growth phases only the short one could be detected. These results indicated that the expression and function of some *Xoo *sRNAs were under strict regulation during the life span of these bacteria.

For the other six sRNAs (sRNA-*Xoo*1, 3, 4, 6, 7, 8), a single major band was detected. Among these six sRNAs, the expression of two sRNAs (sRNA-*Xoo*1, sRNA-*Xoo*3) peaked in the early growth phases, but decreased around entering of the later exponential phase and the entire stationary phase; the other four sRNAs (sRNA-*Xoo*4, sRNA-*Xoo*6, sRNA-*Xoo*7, sRNA-*Xoo*8) showed constant expression levels in different growth phases. Of note, the expression levels of sRNA-*Xoo*4 were dramatically higher than the other sRNAs throughout the growth phases, which was in agreement with its high clone frequency. sRNA-*Xoo*4 possessed distinct self-complementary secondary structure, resembling the SRP (signal-recognition particle) RNA. Such secondary structure may partially contribute to the stable abundance of this sRNA. An sRNA with similar secondary structure was also reported in *Streptomyces coelicolor *[43]. The secondary structures of these identified sRNAs predicted by MFOLD program are showed in Additional file 5.

### Hfq-dependent sRNAs

Hfq is a RNA-binding protein and plays important roles in sRNA functions. Nearly half of the sequenced eubacterial genomes encode an Hfq homolog [21]. Using the Hfq sequence of *E. coli*, we identified a single copy gene encoding highly conserved Hfq protein in the *Xoo *PXO99^A ^genome using BlastP search (score = 135, e-value = 4e-33). To assess whether *hfq *was independently transcribed or transcribed as part of an operon, we performed RT-PCR using primers spanning the two adjacent genes. The result shows that *hfq *is co-transcribed with its adjacent genes (PXO_00156 and PXO_00154) as an operon (Additional file 6).

To investigate the functions of *Xoo *Hfq protein, we constructed a non-polar, in-frame deletion mutant of *hfq *(Δ*hfq*). When cultured in rich PSA medium, the Δ*hfq *mutant showed a longer lag phase and reached the stationary phase at a lower optical density compared with the wild-type strain (Additional file 7), whereas no difference was detected when cultured in minimal medium MMX. Genetic complementation of *hfq *using a recombinant pHM1 plasmid, in which a full-length *hfq *gene was subjected to the control of a p*lac *promoter, restored the growth of Δ*hfq *in PSA medium (Additional file 7). Although deletion of *hfq *in several animal bacteria pathogens resulted in attenuation of virulence [44], mutation in *hfq *in *Xoo *PXO99 did not cause detectable decrease of virulence after inoculation into the host plant rice cultivar IR24. Both the wild-type and Δ*hfq *mutant strains caused blight disease symptoms on rice leaves and had no difference in mean lesion lengths. These results suggested that Hfq is not involved in the virulence of *Xoo *under the experimental conditions used in this study, and its biological roles need to be investigated in further studies.

Successful construction of the *hfq *mutant provided us the opportunity to assess the relationship between Hfq and the confirmed sRNAs in *Xoo*. By Northern blot hybridization, the expression level of the identified sRNAs in the Δ*hfq *mutant was compared to the wild-type strain grown under identical experimental conditions (Figure 2). The expressions of three sRNAs (sRNA-*Xoo*1, 2, 3) were affected in the Δ*hfq *mutant. The transcriptional levels of sRNA-*Xoo*1 and sRNA-*Xoo*3 decreased significantly throughout the growth phases in the Δ*hfq *mutant, indicating that the expression or stability of these sRNAs was completely dependent on Hfq. The sRNA-*Xoo*1 was predicted and annotated as a riboswitch previously [41]. However, based on its altered expression pattern in the Δ*hfq *mutant, we would argue that sRNA-*Xoo*1 is an Hfq-dependent sRNA. Northern blotting of sRNA-*Xoo*2 revealed two unambiguous bands, suggesting a possible presence of post-transcriptional modification of the sRNA-*Xoo*2 primary transcript. The expression profile of sRNA-*Xoo*2 altered dramatically in the Δ*hfq *mutant. The disappearance of the shorter transcript indicates that the putative processing step of sRNA-*Xoo*2 is Hfq-dependent or the shorter form of sRNA-*Xoo*2 is particularly unstable in the Δ*hfq *mutant. The other five sRNAs are likely to be Hfq independent as their expression remained unchanged in the Δ*hfq *mutant. The functions of these Hfq independent sRNAs remain to be elucidated.

### Functional characterization of sRNAs

To investigate the biological functions of these experimentally identified sRNAs, we successfully created sRNA-deleted mutants of the experimentally verified sRNAs (except for sRNA-*Xoo*8) without affecting the expression of the flanking genes. The correct deletion of each sRNAs-coding gene was confirmed by sequencing. These sRNA-deleted mutants were then characterized for changes in phenotype. No significant differences between the ΔsRNA mutants and the wild-type strain were observed in growth rates when the bacteria were grown in both the rich medium (PSA) and minimum medium (MMX), virulence and activities of extracellular enzymes (including extracellular protease, amylase and cellulose). The lack of phenotypic changes of these seven sRNA-deleted mutants may be ascribed to either the minor regulatory roles or the functional redundancy of the identified *Xoo *sRNAs. Further study on these sRNAs by other approaches such as overexpression may provide us some clues into the nature of their functions.

Targets identification is a key step to elucidate the functions of these experimentally verified *Xoo *sRNAs. It has been demonstrated that *trans*-encoded sRNAs usually regulate their target genes via short, discrete and incomplete complementary base pairing, making it difficult to predict the target genes for bacterial sRNAs. In order to identify their potential targets and regulatory roles, three sRNAs (sRNA-*Xoo*1, sRNA-*Xoo*3 and sRNA-*Xoo*4) were selected for further proteomic analysis. Although the proteins identified via two-dimensional gel electrophoresis (2-DE) analysis may either be directly or indirectly regulated by sRNAs, this analysis would provide us some insights to understand the regulatory functions of these sRNAs.

For the proteomic analysis, total proteins from the wild-type strain and sRNA mutants were harvested in the stationary stage (OD_600 _= 1.5) which was consistent with the culture conditions used to clone the sRNA candidates. After 2-DE separation, protein spots that showed more than 1.4-fold change in relative abundance between the wild-type and mutant were selected for mass spectrometry (MS) identification and further analysis. The 2-DE analysis results of the three sRNAs are described separately below.

#### sRNA-*Xoo*1

**Figure 4:**
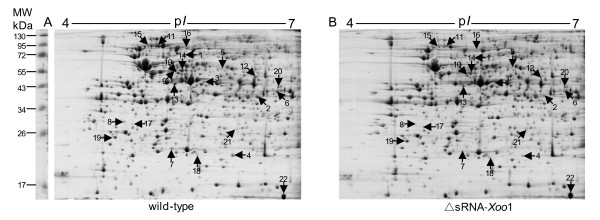
**2-DE maps of total proteins from *Xoo *wild-type strain PXO99 (A) and ΔsRNA-*Xoo*1 mutant (B)**. Protein spots indicated by numbers are the differentially expressed proteins between the wild-type and ΔsRNA-*Xoo*1 mutant. All the labelled spots were identified by MS.

As shown in Figure 3, this Hfq-dependent sRNA is encoded by the intergenic region between PXO_02602 (encoding a transcriptional regulatory factor) and PXO_02603 (*thiC*, encoding a thiamine biosynthesis protein). Based on the distinct expression pattern of sRNA-*Xoo*1 in the D*hfq *mutant and the changes in expression levels during different growth phases in the wild-type strain, we believe that sRNA-*Xoo*1 has important regulatory functions. The 2-DE maps of the DsRNA-*Xoo*1 mutant and wild-type strain are shown in Figure 4. The detailed information on the differentially expressed proteins including functional categories is listed in Table 4.

**Table 4:** Differentially expressed proteins in ΔsRNA-*Xoo*1 identified by MS

Spot ID	protein name	functions	**NCBI acc.no**.	mascot score	Sequence coverage	theoretical MW(Da)/p*I*	Ratio ± SD
**Down-regulated protein spots in sRNA-*Xoo*1 mutant**							
B1	putative ABC transporter ATP-binding protein	transport	PXO_04409	448	56%	61740/5.37	11.57 ± 4.83
B2	glyceraldehyde-3-phosphate dehydrogenase	metabolism	PXO_02308	529	69%	36134/6.35	4.75 ± 0.57**
B3	argininosuccinate synthase	amino acid biosynthesis	PXO_00352	769	67%	45217/5.66	3.25 ± 0.56
B4	tryptophan repressor binding protein	amino acid metabolism	PXO_03044	438	52%	20167/6.05	2.83 ± 0.18**
B5	3-isopropylmalate isomerase large subunit	amino acid biosynthesis	PXO_02613	314	42%	51984/5.68	1.86 ± 0.21**
B6	N-acetylornithine carbamoyltransferase	amino acid biosynthesis	PXO_00353	314	65%	37582/6.07	1.7 ± 0.19**
B7	superoxide dismutase	oxidation reduction	PXO_00389	343	68%	22703/5.47	1.42 ± 0.03**
B8	septum site-determining protein MinD	replication	PXO_04464	435	46%	28837/5.21	1.57 ± 0.19**
B9	N-ethylammeline chlorohydrolase	metabolism	PXO_00380	263	62%	48943/5.19	ND
B10	2-isopropylmalate synthase	amino acid biosynthesis	PXO_02609	694	71%	53421/5.54	1.54 ± 0.15**
B11	translation elongation factor G	transcription and translation	PXO_04525	642	58%	75924/5.09	4.82 ± 0.42**
B12	dihydrolipoamide dehydrogenase	amino acid metabolism	PXO_01196	582	67%	50445/5.98	2.29 ± 0.54
B13	N-acylglucosamine 2-epimerase	metabolism	PXO_01231	811	77%	46884/5.27	2.23 ± 0.34**
B14	ATP synthase subunit alpha	transport	PXO_03111	777	48%	55391/5.38	2.23 ± 0.5
B15	phosphoenolpyruvate synthase	Pyruvate metabolism	PXO_00922	622	48%	86689/5.16	6.36 ± 1.69
B16	Polyribonucleotide nucleotidyltransferase	metabolism	PXO_01307	729	55%	75502/5.47	5.39 ± 0.93
**Up-regulated protein spots in ΔsRNA-*Xoo*1 mutant**							
B17	pyrroline-5-carboxylate reductase	amino acid metabolism	PXO_01991	301	51%	29364/4.93	1.48 ± 0.07**
B18	peptide deformylase	protein synthesis	PXO_04055	379	55%	17129/5.29	1.45 ± 0.02**
B19	outer membrane protein	transport	PXO_03097	343	32%	23707/5.27	1.43 ± 0.04**
B20	Chaperone protein dnaJ	DNA replication	PXO_01186	702	64%	41079/6.21	1.52 ± 0.14**
B21	hydrolase, carbon-nitrogen family	metabolism	PXO_06060	526	68%	30229/5.89	1.57 ± 0.12**
B22	hypothetical protein	hypothetical protein	PXO_01766	635	75%	16502/6.3	1.41 ± 0.01

ND: not detectable in the 2-DE maps of wild-type or ΔsRNA-*Xoo*1 mutant. SD: Standard Deviation. Statistical significance was determined using Student's two-tailed *t *test for unpaired means *P *< 0.05, *P *< 0.01**.

We identified altogether 6 and 16 proteins that were up or down-regulated in the DsRNA-*Xoo*1 mutant, respectively (Table 4). Two important aspects can be defined from these differentially expressed proteins. (1) Most of these proteins participate in amino acid metabolism. Among them, it is notable that the phosphoenolpyruvate (PEP) synthase was down-regulated 6.36-fold compared to the wild-type. PEP is involved in important metabolic pathways such as glycolysis and gluconeogenesis, and in synthesis of chorismate through the shikimate pathway, which is critical for biosynthesis of aromatic amino acids such as phenylalanine, tryptophan and tyrosine. Thus, we concluded that sRNA-*Xoo*1 may be a positive regulator of aromatic amino acids synthesis through direct or indirect promotion of PEP synthase activity. (2) Proteins related to material transport were also regulated in the DsRNA-*Xoo*1 mutant. The most substantially down-regulated protein was a putative ABC transporter that can transport proteins, polysaccharides or low-molecular weight materials, suggesting that sRNA-*Xoo*1 is also associated with secretion, although the secreted substrate remains unknown.

#### sRNA-*Xoo*3

This Hfq-dependent sRNA is expressed from the IGR which is 353 bp in length. Two genes (PXO_03613 and PXO_03614) flanking sRNA-*Xoo*3 are in different orientations. The expression levels of this sRNA changed according to different growth phases in the wild-type and dramatically decreased in the Δ*hfq *mutant, suggesting that it harbours important regulatory functions.

The 2-DE map of the ΔsRNA-*Xoo*3 mutant is shown in Additional file 8A. From this mutant, 18 proteins with decreased expression and eight proteins with enhanced expression were identified. The down-regulated proteins can be classified into three major groups: oxidation reduction; response to stress and metabolism (Additional file 9). Of note, seven proteins including dehydrogenases, monooxygenase, reductase and superoxide dismutase which are related to the process of oxidation reduction were down-regulated in the DsRNA-*Xoo*3 mutant. Consistent with this observation, a protein related to ATP synthesis was up-regulated to 2.97 fold in the DsRNA-*Xoo*3 mutant. We hypothesized that this sRNA plays roles in regulating energy metabolism which is essential for the organism. The other seven up-regulated proteins in the DsRNA-*Xoo*3 mutant were assigned to various metabolic functions, including glutathione metabolism, fatty acid metabolism, lipopolysaccharide, and sucrose metabolism.

#### sRNA-*Xoo*4

The sRNA-*Xoo*4 is positioned in the IGR between PXO_05774 and PXO_02847 which is 184 bp in length, and the 5' end of sRNA-*Xoo*4 was mapped to the 3' end of the upstream gene PXO_05774, a hypothetical protein of unknown function. The lack of the promoter motifs in the upstream region of the sRNA-*Xoo*4 suggests that it may be processed from the upstream gene. The dramatically high expression levels and the specific single hairpin secondary structure suggested that sRNA-*Xoo*4 has some important functions.

The 2-DE map of the DsRNA-*Xoo*4 mutant is also shown in Additional file 8B. In the DsRNA-*Xoo*4 mutant, nine down-regulated proteins mainly impaired DNA replication, rRNA processing and metabolic processes. Since the inactivation of this sRNA affected the expression of genes involved in DNA replication, transcription and translation, it may be involved in some house-keeping like functions. Nine proteins with enhanced expression in the DsRNA-*Xoo*4 mutant mainly took part in transport and protein secretion. Membrane proteins such as the TonB receptor and outer membrane (OM) protein were up-regulated in the DsRNA-*Xoo*4 mutant, indicating that its function may also be related to material transport. Among these up-regulated proteins, a fimbrial assembly membrane protein (PXO_02354) was up-regulated 1.93-fold comparing with the wild-type. Fimbriae are filamentous appendages on the bacterial surface, which are anchored within the OM, regulating the protein secretion via close contact with the host cells. This result indicated that DsRNA-*Xoo*4 may play a role in pathogen-host recognition. Detailed information on the differentially expressed proteins is listed in Additional file 9.

In brief, the 2-DE results provided experimental evidence that the above three sRNAs in *Xoo *are involved in multiple physiological and biochemical processes. Because it was reported that one *trans*-encoded sRNA usually has many targets, it is possible that proteins identified by 2-DE analysis may be directly regulated by the sRNA. However, indirect regulations could occur in the null mutant of sRNA. Further analysis should clarify which genes are the real and direct targets of the sRNAs. Therefore, the differentially expressed proteins characterized by the 2-DE approach in this work were useful for providing potential targets of the identified sRNAs in *Xoo*, and these findings provide the initial step toward dissecting their functions.

### Analysis of *cis*-encoded sRNA candidates

The detection of the 2 cloned sequences without sRNA prediction support by Northern blotting suggested that our sRNA selection criteria were too strict and had excluded some authentic sRNAs. If consider all cloned non-coding short RNA sequences as sRNA candidates, a total of 180 non-redundant putative *cis*-encoded sRNAs were cloned. Recent transcriptomic analyses have revealed that *cis*-encoded antisense sRNAs exist generally in bacterial cells, albeit their metabolic processes and functional roles remain to be investigated [29]. Among the 180 non-redundant putative *cis*-encoded sRNAs candidates in our library, only 16 (8.9%) were generated by transposons or insertion elements, whereas all the other candidates were transcribed from non-transposable regions. Although the full-length sequences of these sRNA candidates were unclear, alignments showed that 108 of them were complementary to the central regions of potential target mRNAs; 44, 16 and 12 sequences were complementary to, respectively, the 3' region, the 5' region or the entire sequence of their target mRNAs and overlapped with the adjacent UTRs (Additional file 10). The difference in base pairing locations may reflect the distinct regulatory mechanisms of these putative sRNAs.

As revealed in other bacteria, *cis*-encoded antisense RNA is also prevalent [30]. For example, in the human gastric pathogen *Helicobacter pylori*, massive parallel cDNA sequencing found that 46% of all ORFs have at least one antisense transcriptional start site [45]. Therefore, the identification of such a large number of *cis*-encoded antisense sRNA candidates in *Xoo *suggested that this type of sRNA may take part in regulating various cellular processes. The putative target genes of the above mentioned *cis*-encoded sRNA candidates could be classified into diverse functional categories (Additional file 10). Eight TonB-dependent receptor genes were predicted to be targeted by *cis*-encoded antisense sRNAs, constituting the largest functional group among the virulence-associated genes. TonB-dependent receptor genes are required for the bacterial uptake of iron that is usually limited in the host organism [46], therefore play an important role for the survival of bacteria within host tissues or cells [47]. In previous studies, it was revealed that several TonB-dependent receptors, such as IroN, BfeA and BtuB are involved in pathogenesis of *Xcc*, supporting that iron-uptake is also critical for the pathogenicity of *Xanthomonas *spp. [48,49]. However, in *Xoo*, aside from the regulatory protein Fur (ferric iron uptake regulator) [50], little is known about how the iron-uptake systems are modulated and involved in virulence. In *E. coli*, the small RNA RyhB has been reported to be essential for iron metabolism [51,52] and intracellular iron homeostasis by regulating genes responsible for siderophore and cysteine biosynthesis [53,54], providing evidence that small RNA also takes part in regulation of iron uptake in bacteria. Consequently, further study on the function of the above identified TonB-dependent receptors and the corresponding *cis*-encoded sRNA candidates will promote our understanding in this field.

## Conclusions

Through cloning and bioinformatic methods in the present study, eight sRNAs in *Xoo *were experimentally confirmed among a large number of sRNA candidates, three of which were determined to be Hfq-dependent sRNAs. We provided experimental evidence to clarify that these sRNAs play roles in diverse important biological pathways in *Xoo*. Although the direct targets will need to be identified to further dissect the function of these sRNAs, the differentially expressed proteins revealed by proteomic analysis in our study provided valuable hints and served as a starting point for unraveling the sRNA mediated regulatory networks in *Xoo*.

## Methods

### Bacterial strains and culture conditions

All bacterial strains used in this study are listed in Additional file 11. *Xanthomonas oryzae *pv. *oryzae *PXO99 was routinely cultured in rich medium PSA (tryptone, 10 g/L; sucrose, 10 g/L; glutamic acid, 1 g/L; pH 7.0) or in minimal medium MMX (sodium citrate, 1.0 g/L; K_2_HPO_4_, 4 g/L; KH_2_PO_4_, 6 g/L; (NH_4_)_2_SO_4_, 2 g/L; MgSO_4_·7H_2_O, 0.2 g/L; and glucose, 5 g/L; pH 7.0) at 28°C. *E. coli *strain DH10B™was used in library and plasmid constructions, and clones were grown in Luria-Bertani broth at 37°C. Antibiotics were added to media when required at final concentrations of 50 μg/mL of kanamycin for both *Xoo *and *E. coli*; and at 100 μg/mL of ampicillin for *E. coli*.

### Construction and analysis of the cDNA library

The methods used to clone the small RNAs in this study was based on a published protocol [55]. Cultures of *Xoo *were harvested at OD_600 _of 1.5. Total RNA was extracted using the TRIzol reagent (Invitrogen), and 300 μg RNA pre-treated with RQ1 RNase-free DNase I (Promega) was separated on denaturing 8% polyacrylamide gels (7 M urea). RNAs ranging from 50 to 500 nt were eluted and ethanol precipitated [56]. The size selected RNAs were 3' tailed by CTP and poly (A) polymerase and then reverse transcribed by oligo (G) primer (see Additional file 12 for sequence) and SuperScript™II Reverse Transcriptase (Invitrogen). Subsequently, cDNAs were cloned into the pSPORT1 vector (Invitrogen) and transformed into *E. coli *strain DH10B™competent cells.

Initially, 192 clones were sequenced, which allowed us to identify the abundant rRNA and tRNA fragments in the library. Specific probes (Additional file 12 for sequence) were designed based on these sequences to deplete the highly abundant RNA species. Pre-screening was performed as follows: cDNA inserts of individual clones were amplified using the M13 forward and M13 reverse primers, and then the PCR products were purified and spotted in high-density arrays. Hybridization was performed using specific probes. Clones showing the lowest signals were selected to sequence. Bioinformatic analysis was performed by first excluding sequences representing rRNA, tRNA and sRNAs reported in Rfam. The remaining sequences were then mapped to the *Xoo *PXO99^A ^genome by BLAST searches. The putative sRNA genes were further categorized based on the strands and positions relative to the flanking ORFs. Non-coding RNA structures were predicted using MFOLD version 3.0. VIMSS operon prediction was used to predict the operon organization in *Xoo *PXO99^A^. Sequences for sRNA-*Xoo*1-8 were deposited in GenBank with accession numbers from HQ890319 to HQ890326.

### RNA analysis

Total RNA was extracted from liquid cultures of *Xoo *at different OD_600 _values using TRIzol reagent. RNA samples were quantified using a NanoDrop ND-1000 spectrophotometer (Thermo, USA). RNA (30 μg) were denatured for 5 min at 95°C in Gel Loading Buffer II (Ambion), then separated on denaturing 8% polyacrylamide gels and transferred to Hybond-N^+ ^membrane (Amersham) by electro-blotting. RNAs were immobilized to the membrane by UV cross-linking. Before hybridization the membranes were stained with methylene blue to check the integrity of RNA and also ensure uniform loading of RNA. The DNA oligonucleotide probes (see Additional file 12 for sequences) were labelled with [γ-^32^P] ATP using T4 polynucleotide kinase (NEB). Membranes were pre-hybridized in Church and Gilbert buffer [57] at 42°C for 2 hours and hybridized at 42°C for 16 hours. The membranes were washed with 2 × SSC, 0.1% SDS; 1 × SSC, 0.1% SDS and 0.1 × SSC, 0.1% SDS for 20 min at 42°C sequentially. Hybridization signals were visualized on a phosphorimager. Northern blots for all detectable sRNAs were confirmed for three biological repeats. All RNA samples of the wild-type strain and D*hfq *mutant were treated similarly throughout the experimental steps. Thus any differences observed between the wild-type and D*hfq *mutant could be attributed to changes in the expression of the sRNAs.

For reverse transcription (RT)-PCR analysis, 2 μg of total RNA pre-treated with RQ1 RNase-free DNase I (Promega) were reverse transcribed using random hexamer primers and SuperScript™II Reverse Transcriptase (Invitrogen) as described by the manufacturer. For the negative control, the same RNA samples were also incubated in the same system only without reverse transcriptase. All reactions were performed in 25 μL volumes.

For the 5' RACE, mapping of the 5' end was carried out using the FirstChoice RLM-RACE kit (Ambion), following the manufacturer's instructions, but with modifications for bacterial RNA as outlined by Vogel [58]. The PCR products of 5' RACE were cloned into the pGEM-T vector, and then the clones were sequenced and analyzed. Six to ten clones for each 5' RACE analysis were sequenced, and the farthest 5' end was regarded as the 5' end of the sRNA.

### Generation of mutant strains

The *hfq *and sRNA deletion strains were generated using a two-step homologous recombination strategy. The recombinant suicide vector derived from pk18mobsacB [59] was used to delete *hfq *and sRNA coding genes in *Xoo*. Primers containing specific restriction enzyme sites were used to amplify the upstream and downstream fragments flanking *hfq *and sRNAs coding genes are given in Additional file 12. PCR reactions were performed in 50 μL volumes containing 30 ng DNA of *Xoo*, 1 × PCR buffer, 50 μM each dNTPs, 0.2 μM each primer, 1 mM MgSO_4_, and 1 U of KOD-plus- polymerase (TOYOBO). The PCR was carried out using the following thermal cycling profile: 94°C for 4 min, followed by 34 cycles of amplification (94°C for 30 s, 68°C for 1 min) and finally 72°C for 5 min. The PCR products were gel-purified and digested by relevant restriction enzymes, and purified again. The resulting two fragments were inserted into the pk18mobsacB vector digested with the appropriate restriction enzymes to create the suicide vector. The suicide vector was transformed into *E. coli *Top10. All recombinant vectors were verified by sequencing of the inserted fragments. For homologous recombination, the recombinant plasmids were extracted and electroporated into *Xoo *competent cells using a Micro-pulser set at 18 kVcm^-1^, and the pulse time was approximately 0.3 to 0.4 ms. Transformants were first selected on PSA plates containing kanamycin and then on PSA plates containing 10% sucrose to select double cross-over strains. Finally all mutants were verified by multiple PCR analysis and confirmed by sequencing.

### Plant inoculation and extracellular enzyme assays

Two-month-old susceptible rice cultivar IR24 was used as the host plant. Bacteria were cultured to the early exponential phase, and the OD_600 _value was adjusted to 0.4. The leaves were clipped using sterile scissors which were dipped in the bacteria cultures. Lesion length was scored two weeks after inoculation as described by Dow [60]. The activities of three extracellular enzymes (protease, amylase, cellulose) were analyzed based on previously published methods [61]. PSA plates containing 1.5% skimmed milk, 0.1% soluble starch or 0.5% sodium carboxymethyl cellulose were used to test the protease, amylase and cellulose, respectively. Strains were cultured to OD_600 _of 0.1, and then 1 μL of the cultures were spotted onto the above mentioned plates. The plates were incubated at 28°C for 3-5 days before measuring the enzyme activities following the methods described by Tang *et al*. [61].

### Protein 2-DE analysis

Bacterial cells were harvested and centrifuged at 8,000 rpm for 5 min at 4°C. The cell pellets were washed twice with ice cold 10 mM Tris (pH 7.4) buffer with 250 mM sucrose. The cell pellets were resuspended with 4 mL 10 mM Tris (pH 7.4) buffer containing PMSF (40 μL, 100 mM) and then sonicated for 15 min (cycles of 3 s on and 30 s off). Protein samples were then purified by phenol saturated with Tris-HCl (pH 8.6) and precipitated with five volumes of 0.1 M ammonium acetate in methanol at -20°C overnight. After centrifugation at 12,000 rpm for 20 min at 4°C, the pellet was rinsed twice with ice-cold 0.1 M ammonium acetate in methanol and twice with ice-cold 80% acetone. The air-dried pellet was resuspended in isoelectric focusing (IEF) buffer containing 7 M urea, 2 M thiourea, 4% CHAPS, 40 mM DTT, 2% (v/v) IPG buffer (pH 4-7). Protein concentration was determined using a 2-D Quant kit (GE Healthcare Life Sciences, NJ, USA). The supernatant containing the soluble protein fraction was immediately subjected to 2-DE for protein separation.

The 2-DE procedure was conducted according to a previously published protocol [62]. To obtain the highest possible resolution, 1 mg proteins extracted from the related strains were separated by 2-DE using a nonlinear pH 4-7 IPG strips (24 cm) and SDS-PAGE, and each sample was analyzed in triplicate. Approximately 1,000 ± 50 spots could be detected on each gel. Protein amounts of the detected spots on normalized gels were quantified with ImageMaster 2D Platinum software, and the average intensities of the spots were measured.

Protein spots were excised from gels and washed twice with 400 μL of 50 mM NH_4_HCO_3 _in 50% (V/V) ACN for 15 min to destain. The solution was then removed and 400 μL 100% ACN was added to dehydrate the gel pieces. The gel pellets were vacuum dried and then rehydrated with 3 μL 50 mM NH_4_HCO_3 _containing 20 ng/μL trypsin (Promega) at 4°C for 45 min. An additional 3 μL of 50 mM NH_4_HCO_3 _was added, and the reaction was incubated at 37°C overnight. The liquid was removed to a fresh tube, and the gel pellet was extracted again with 0.1% TFA in 50% ACN at 37°C for 1 hour. The liquid was again transferred to a fresh tube, and the liquid containing the peptides was finally vacuum dried and analyzed by MS. The detailed methods were performed as described in a previous study [62].

The identified differentially expressed proteins were functionally categorized by using the Gene Ontology Tool. The Go enrichment analysis was performed using GOEAST [63].

## Authors' contributions

HL carried out the experiments, analyzed the primary data and wrote the draft manuscript; YTZ performed the bioinformatic related analysis; JQZ participated in clone construction and 2-DE, XJW, RXF and YTJ assisted with experimental design, data analysis, supervised the whole work and revised the manuscript. All authors have read, approved and made contributions to the final manuscript.

## Supplementary Material

Additional file 1 **Outline of procedures used in cDNA library analysis (pdf)**. Flowchart of the steps used for the cDNA library analysis. Each step is shown on the left, and the corresponding number is listed on the right. Click here for file

Additional file 2 **The detailed information of the sequences from the cDNA library (xls)**. Click here for file

Additional file 3 **List of the predicted sRNA candidates in *Xoo *PXO99^A ^using SIPHT search and the comparison of the results of the cloned sequences with the prediction results (xls)**. Click here for file

Additional file 4 **5' RACE results (xls)**. Click here for file

Additional file 5 **Predicted secondary structure of *Xoo *sRNAs (pdf)**. Secondary structures of eight *Xoo *sRNAs were predicted using MFOLD program. Click here for file

Additional file 6 **RT-PCR confirmation of the transcriptional unit of the *hfq *gene (pdf)**. (A) The position and direction of ORFs were presented by arrows, and the corresponding names for each ORFs were also showed. The locations of primers used in the following PCR were presented by arrows. (B) RNAs prepared from wild-type cells cultured in rich (R) and minimum (M) medium were used for the reverse transcription using random primers to synthesis cDNA separately. DNA, positive control; +, with reverse transcriptase; -, without reverse transcriptase (a negative control to show no contamination of genomic DNA in the RNA sample). Click here for file

Additional file 7 **Growth characteristics of the Δ*hfq *mutant in rich medium (pdf)**. OD_600 _values of triplicate cultures in PSA medium were determined in two hour intervals (diamonds: wild-type; squares: Δ*hfq*; triangles: Δ*hfq*-C, *hfq *complementary strain). Click here for file

Additional file 8 **2-DE map of the total proteins from wild-type and the sRNA-deleted mutant strains (pdf)**. (A) 2-DE maps of total proteins from *Xoo *wild-type strain and ΔsRNA-*Xoo*3 mutant. (B) 2-DE maps of total proteins from *Xoo *wild-type strain and ΔsRNA-*Xoo*4 mutant. Protein spots indicated by numbers are the differentially expressed proteins. All these spots were identified by MS. Click here for file

Additional file 9 **Differentially expressed proteins in ΔsRNA-*Xoo*3 and ΔsRNA-*Xoo*4 identified by MS (pdf)**. Click here for file

Additional file 10 **The distribution of *cis*-encoded sRNA candidates and the functional classification of their putative target genes (xls)**. Click here for file

Additional file 11 **Strains and plasmids used in this study (pdf)**. Click here for file

Additional file 12 **Oligonucleotides used in this study (pdf)**. Click here for file
