# Supplementary figures and images for: Identification and functional characterization of small non-coding RNAs in Xanthomonas oryzae pathovar oryzae
Source: BMC Genomics. 2011 Jan 30;12:87. doi: 10.1186/1471-2164-12-87 (PMC3039613; doi:10.1186/1471-2164-12-87)

## Additional file 1

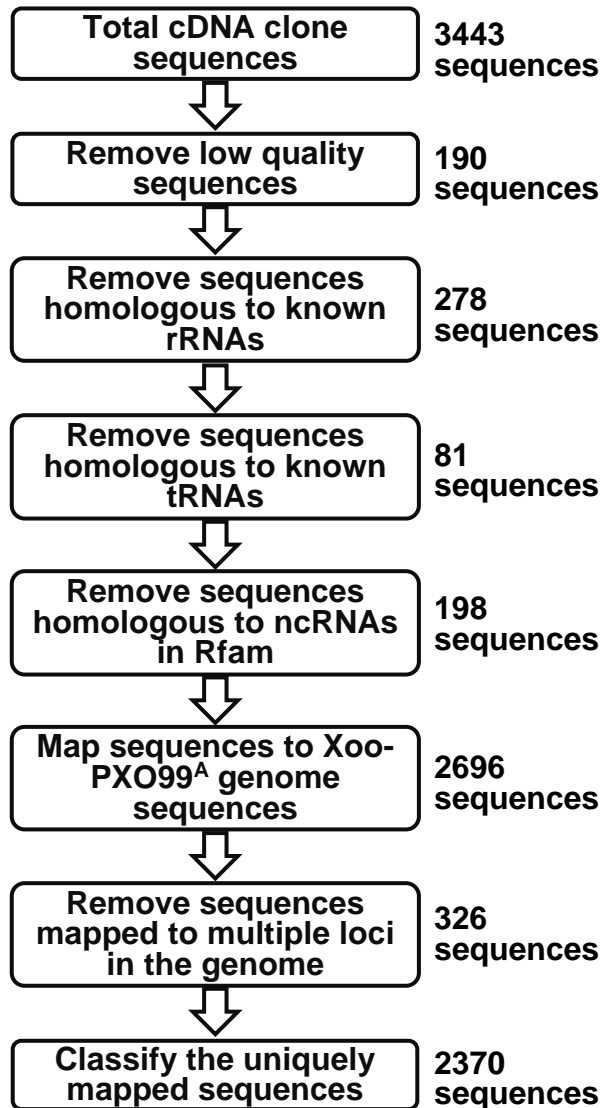

Supplement: Additional file 1 — Outline of procedures used in cDNA library analysis (pdf). Flowchart of the steps used for the cDNA library analysis. Each step is shown on the left, and the corresponding number is listed on the right. [file 1471-2164-12-87-S1.PDF]

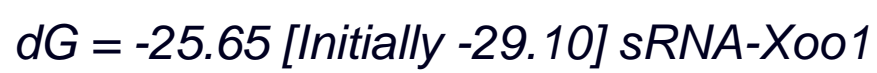

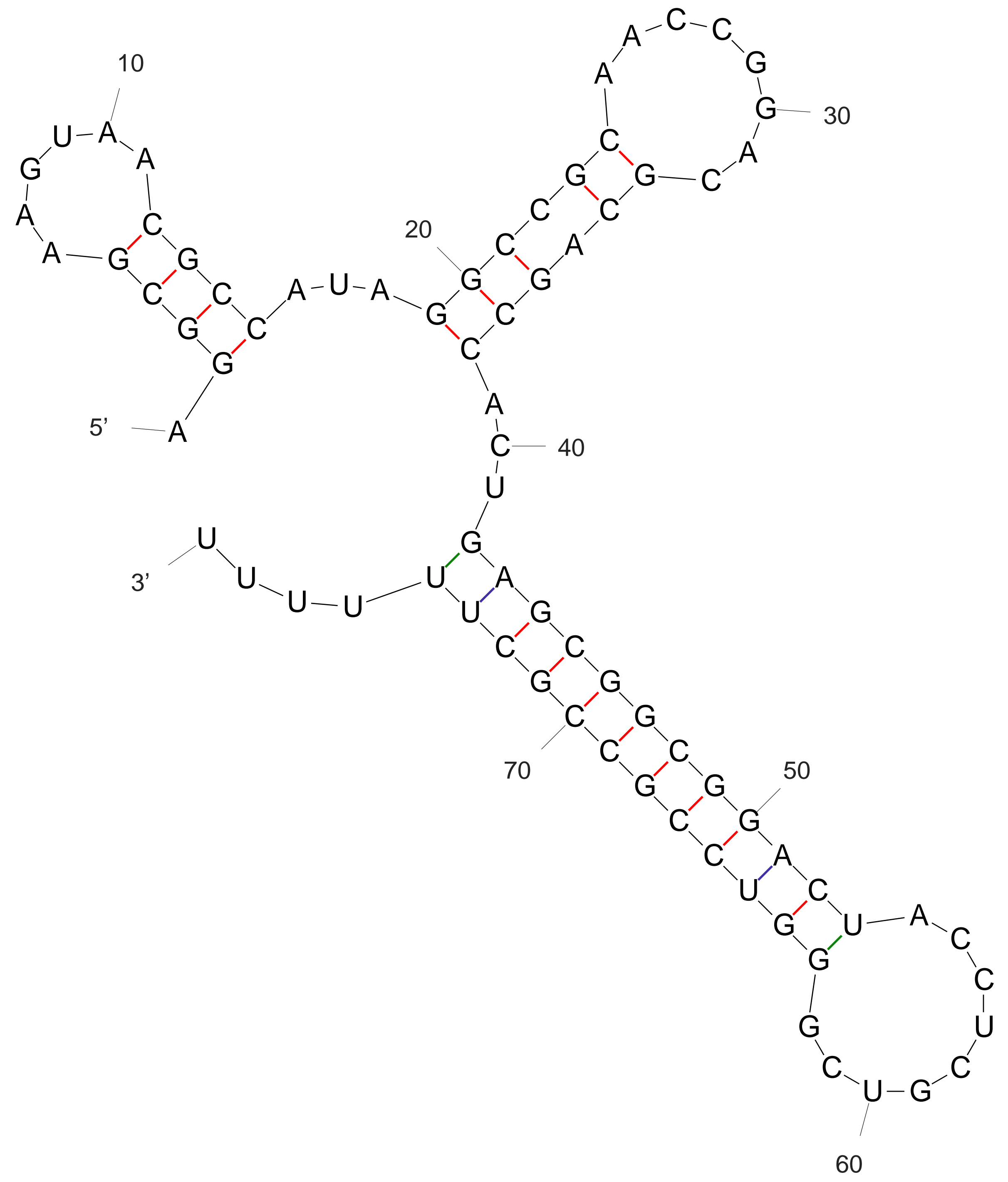

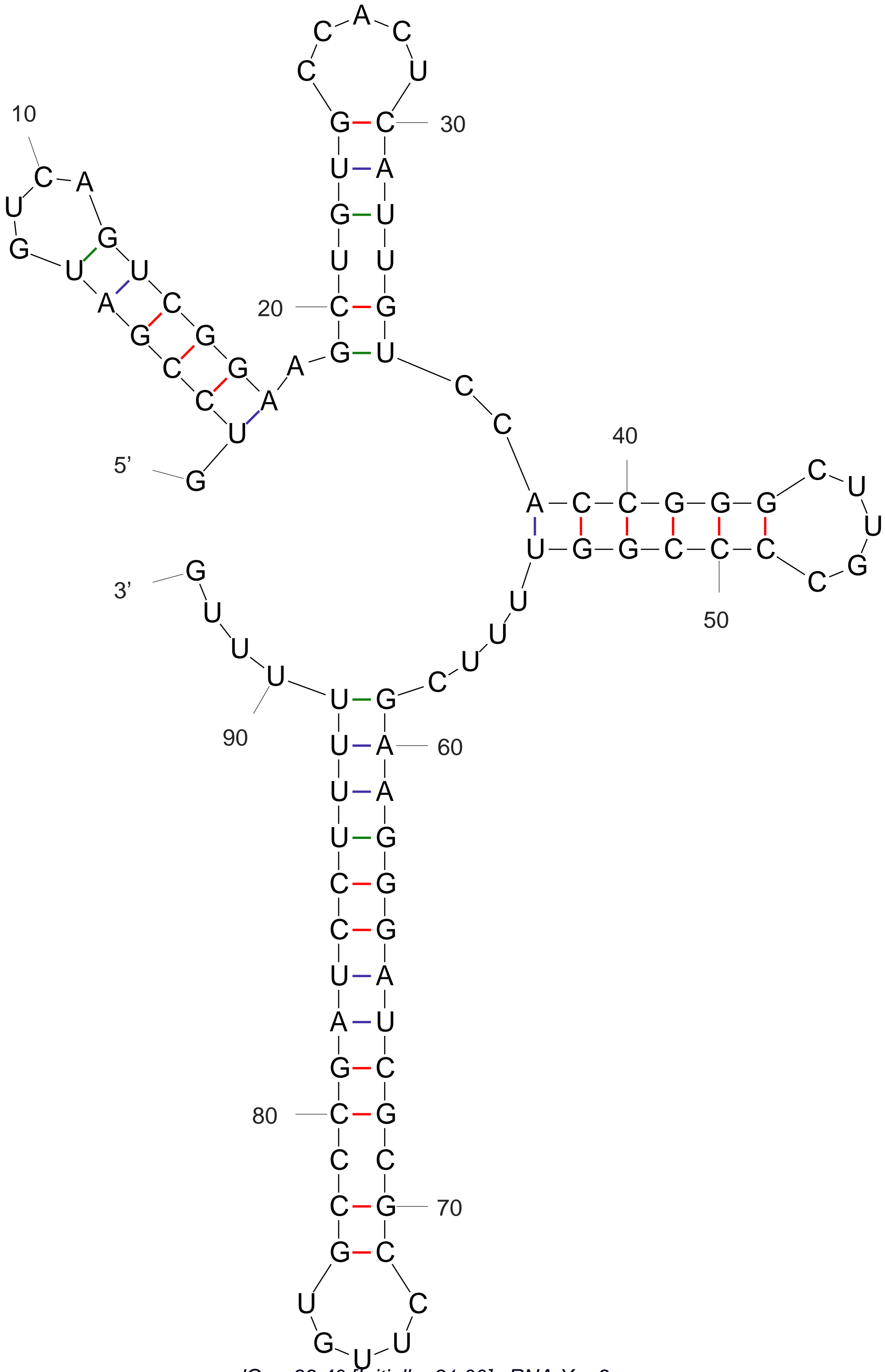

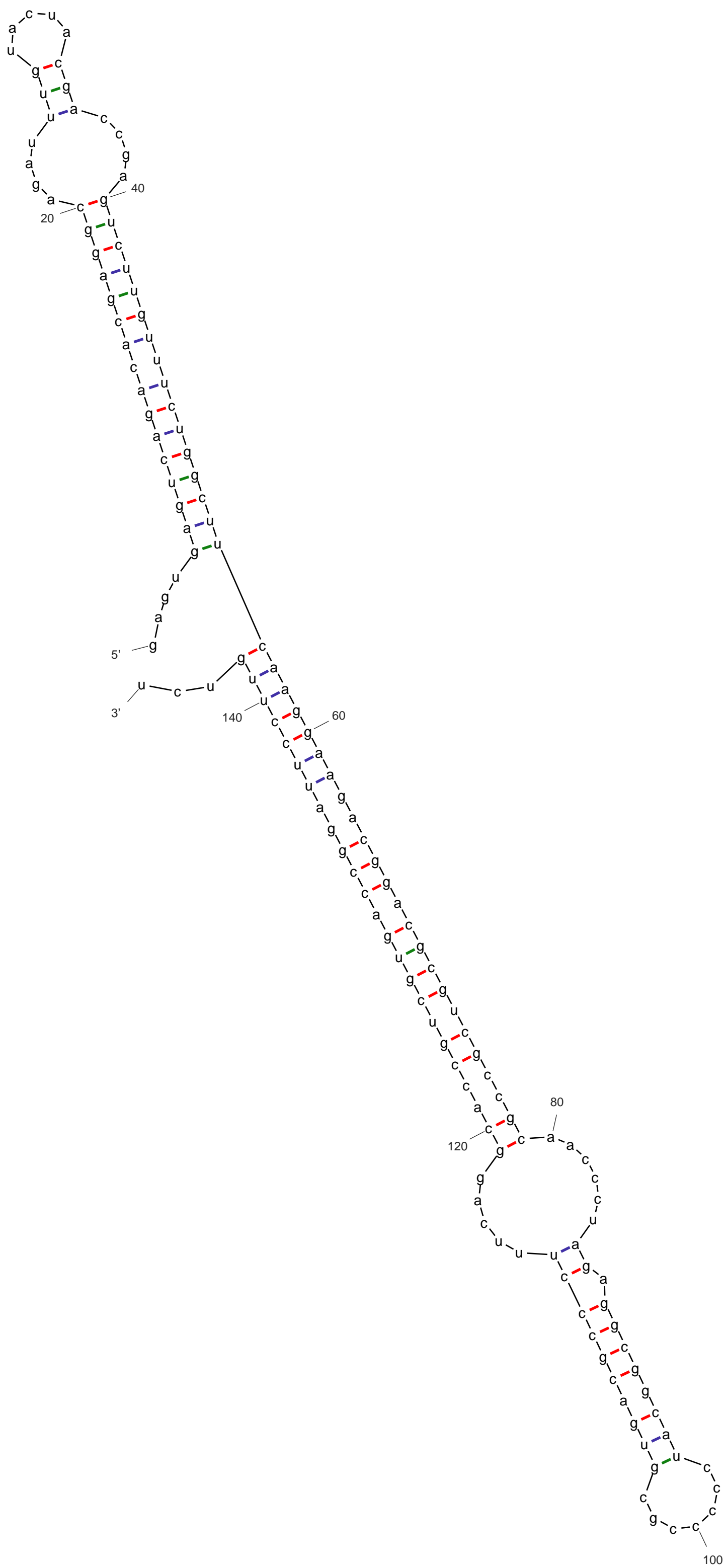

$dG = -55.30$  [Initially -54.60] sRNA-Xoo4

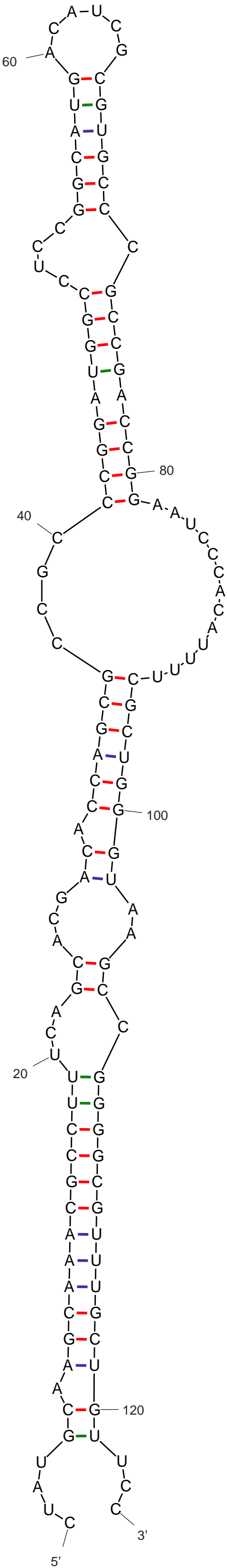

*dG = -51.10 [Initially -51.10] sRNA-Xoo5*

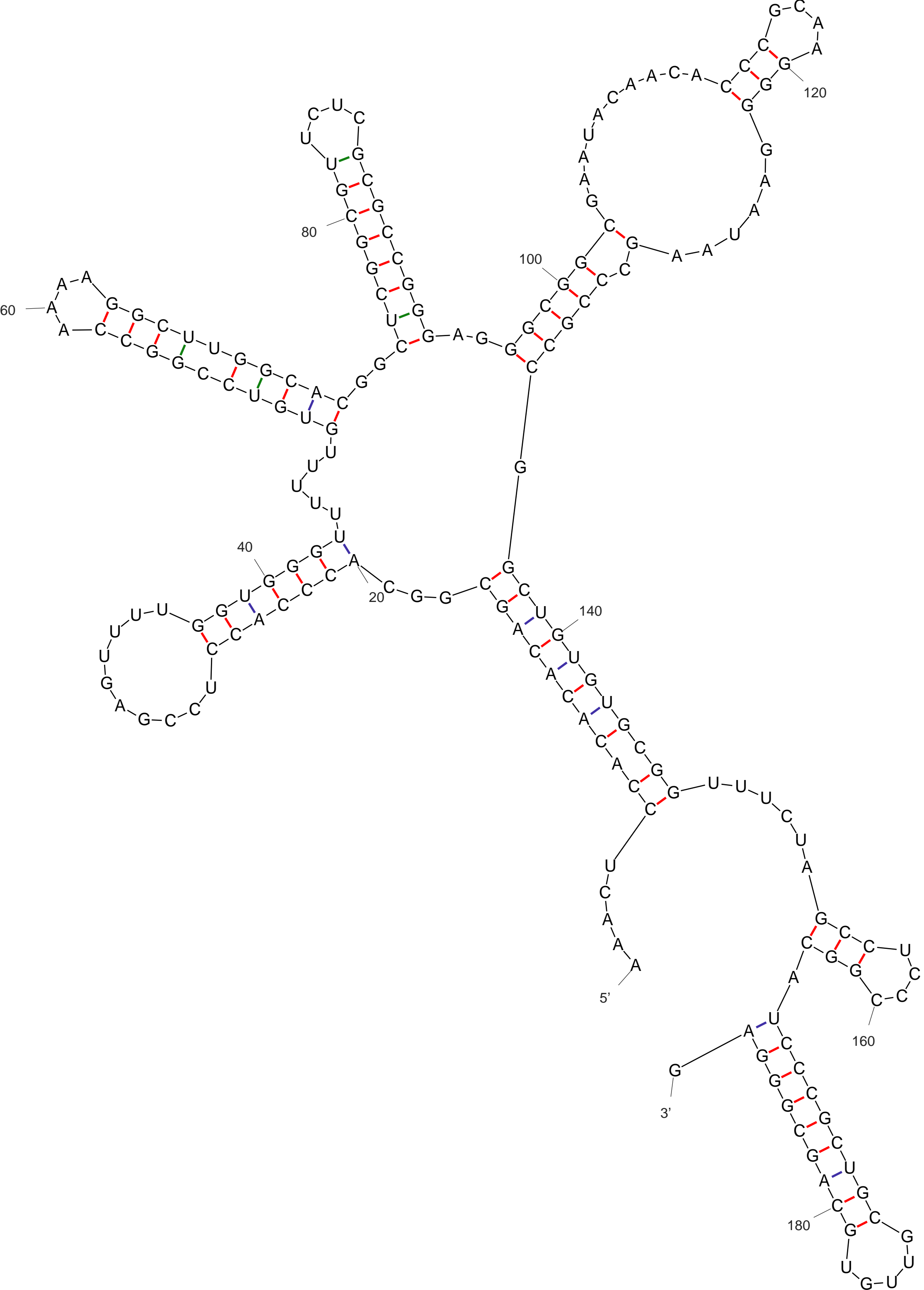

*dG = -93.65 [Initially -95.40] sRNA-Xoo6*

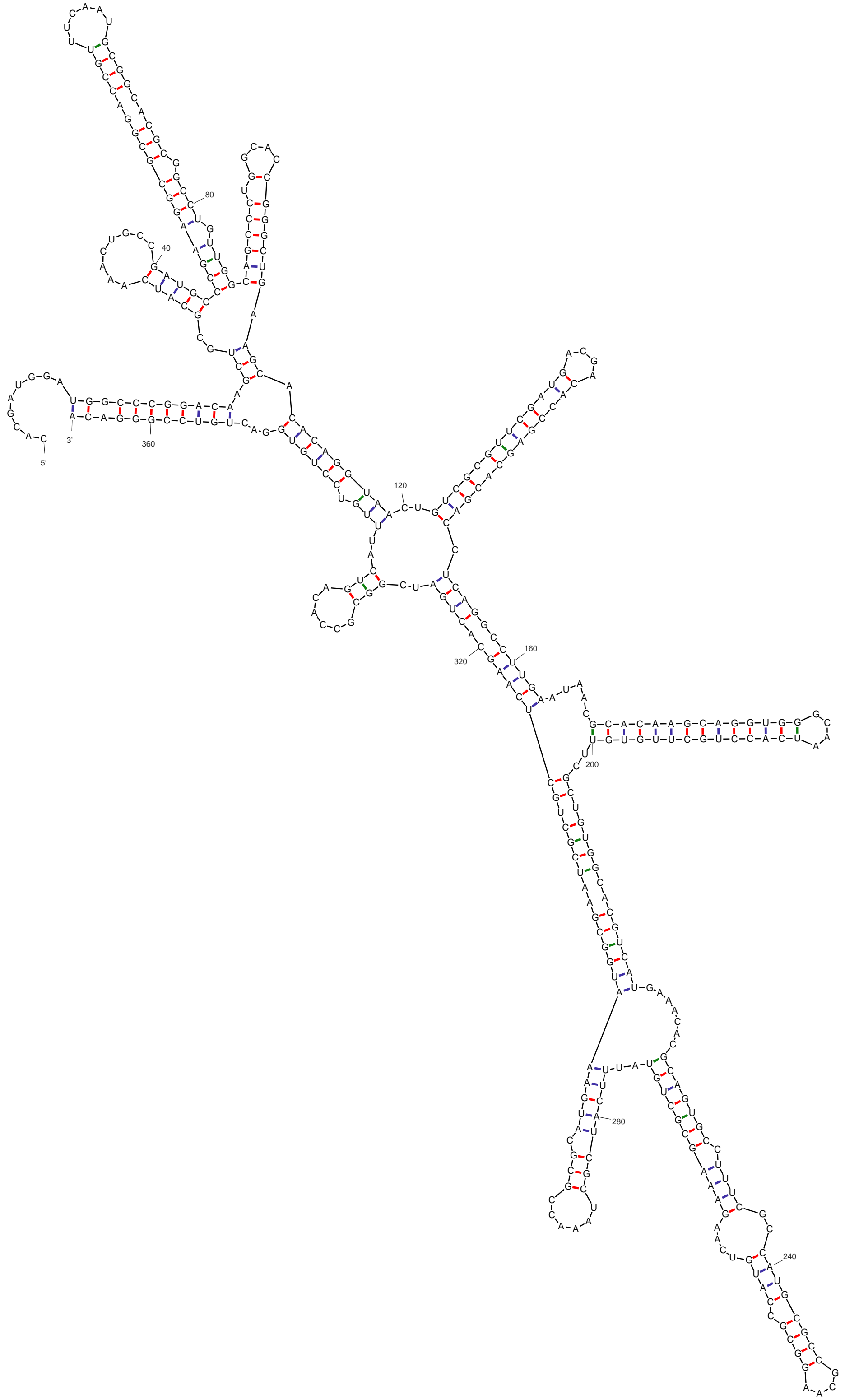

*dG = -155.21 [Initially -160.90] sRNA-Xoo7*

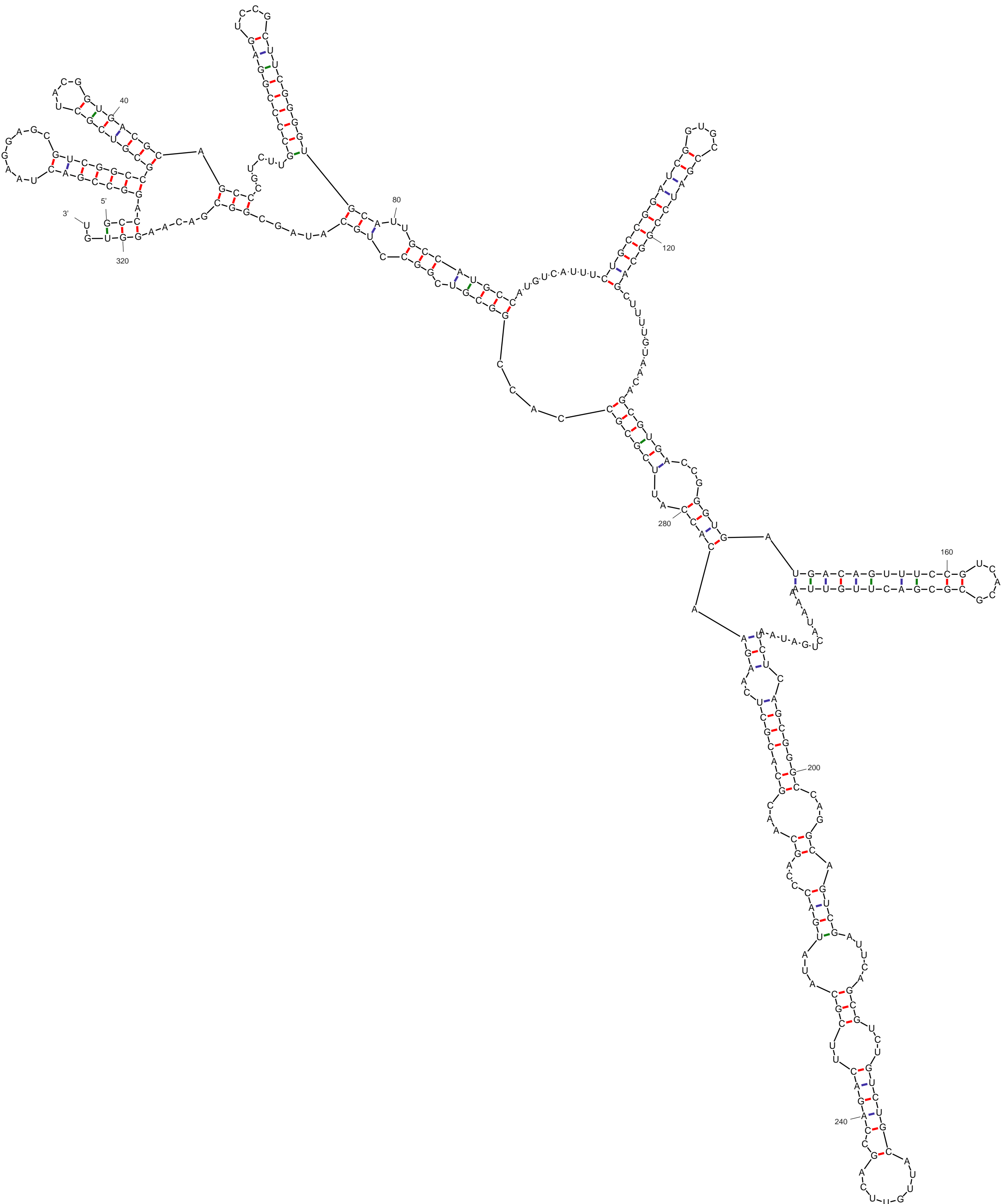

Supplement: Additional file 5 — Predicted secondary structure of Xoo sRNAs (pdf). Secondary structures of eight Xoo sRNAs were predicted using MFOLD program. [file 1471-2164-12-87-S5.PDF]

Additional file 6

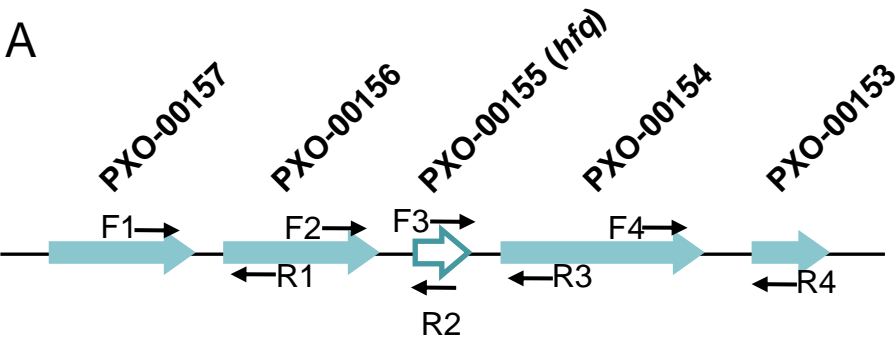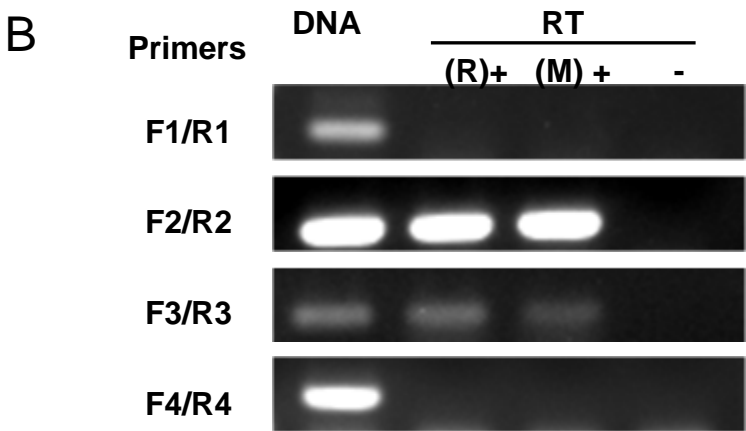

Supplement: Additional file 6 — RT-PCR confirmation of the transcriptional unit of the hfq gene (pdf). (A) The position and direction of ORFs were presented by arrows, and the corresponding names for each ORFs were also showed. The locations of primers used in the following PCR were presented by arrows. (B) RNAs prepared from wild-type cells cultured in rich (R) and minimum (M) medium were used for the reverse transcription using random primers to synthesis cDNA separately. DNA, positive control; +, with reverse transcriptase; -, without reverse transcriptase (a negative control to show no contamination of genomic DNA in the RNA sample). [file 1471-2164-12-87-S6.PDF]

## Additional file 7

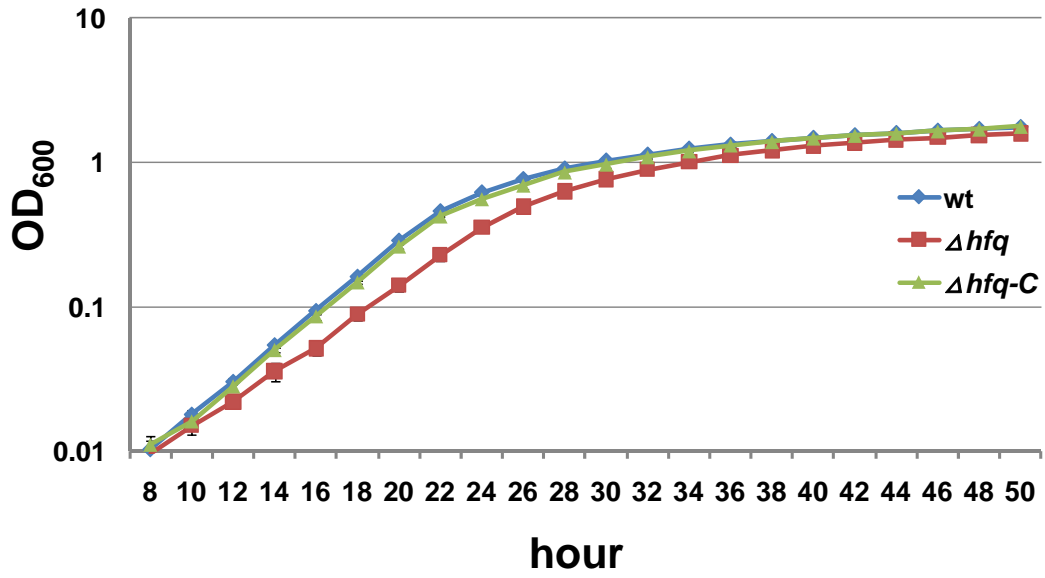

Supplement: Additional file 7 — Growth characteristics of the Δhfq mutant in rich medium (pdf). OD600 values of triplicate cultures in PSA medium were determined in two hour intervals (diamonds: wild-type; squares: Δhfq; triangles: Δhfq-C, hfq complementary strain). [file 1471-2164-12-87-S7.PDF]
