# Supplementary material for: Identification and functional characterization of small non-coding RNAs in Xanthomonas oryzae pathovar oryzae
Source: BMC Genomics. 2011 Jan 30;12:87. doi: 10.1186/1471-2164-12-87 (PMC3039613; doi:10.1186/1471-2164-12-87)
Supplement: Additional file 8 — 2-DE map of the total proteins from wild-type and the sRNA-deleted mutant strains (pdf). (A) 2-DE maps of total proteins from Xoo wild-type strain and ΔsRNA-Xoo3 mutant. (B) 2-DE maps of total proteins from Xoo wild-type strain and ΔsRNA-Xoo4 mutant. Protein spots indicated by numbers are the differentially expressed proteins. All these spots were identified by MS. [file 1471-2164-12-87-S8.PDF]

Additional file 8A

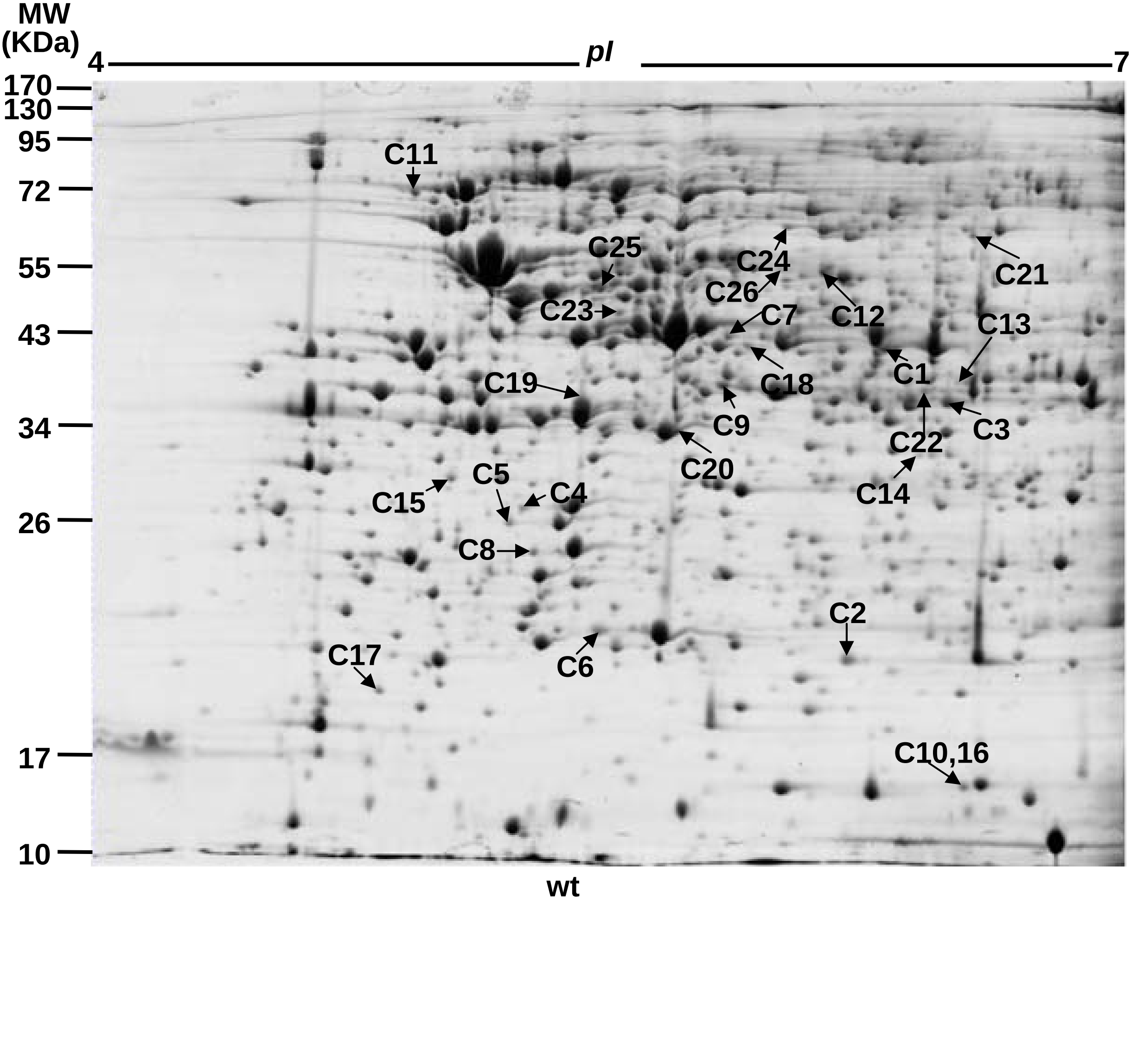

Additional file 8A

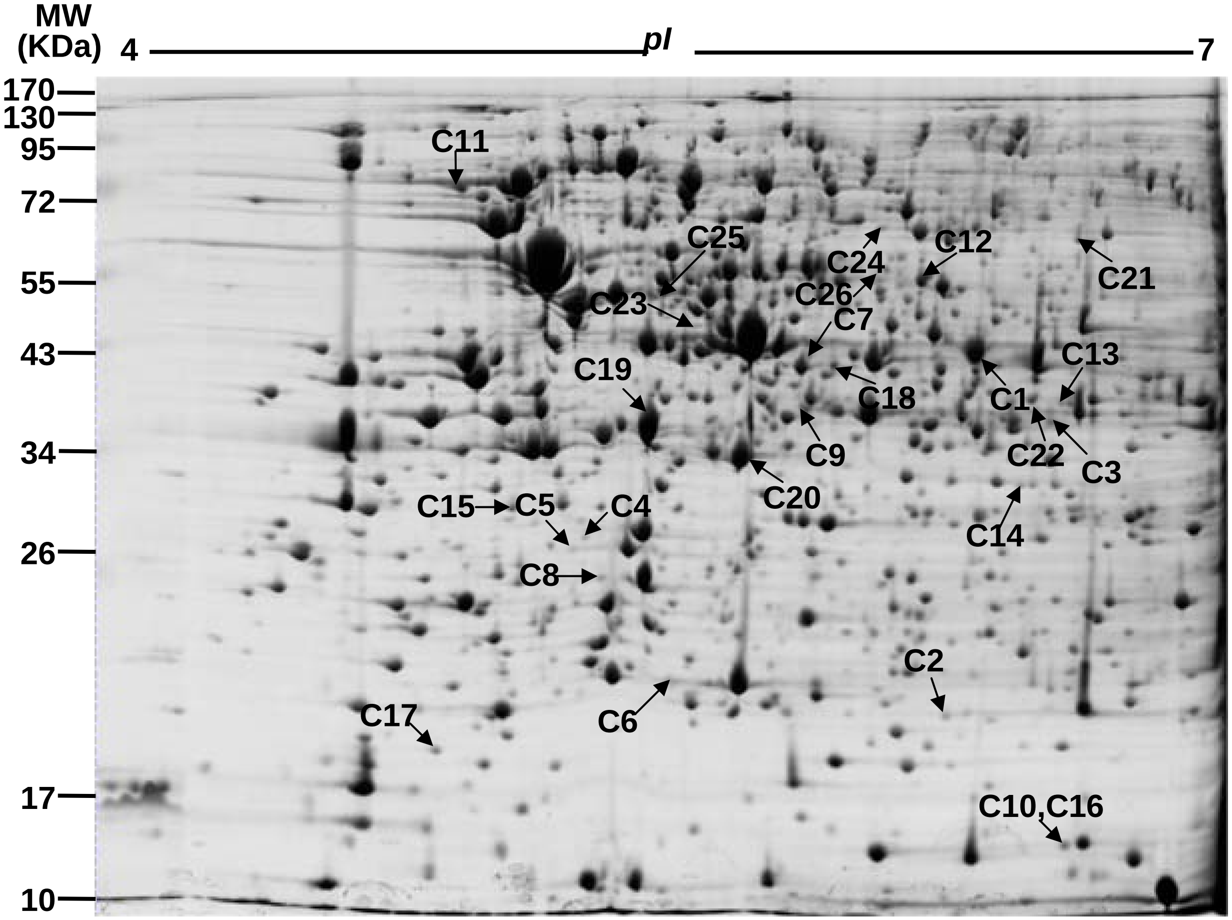

△sRNA-*Xoo*3 mutant

Additional file 8B

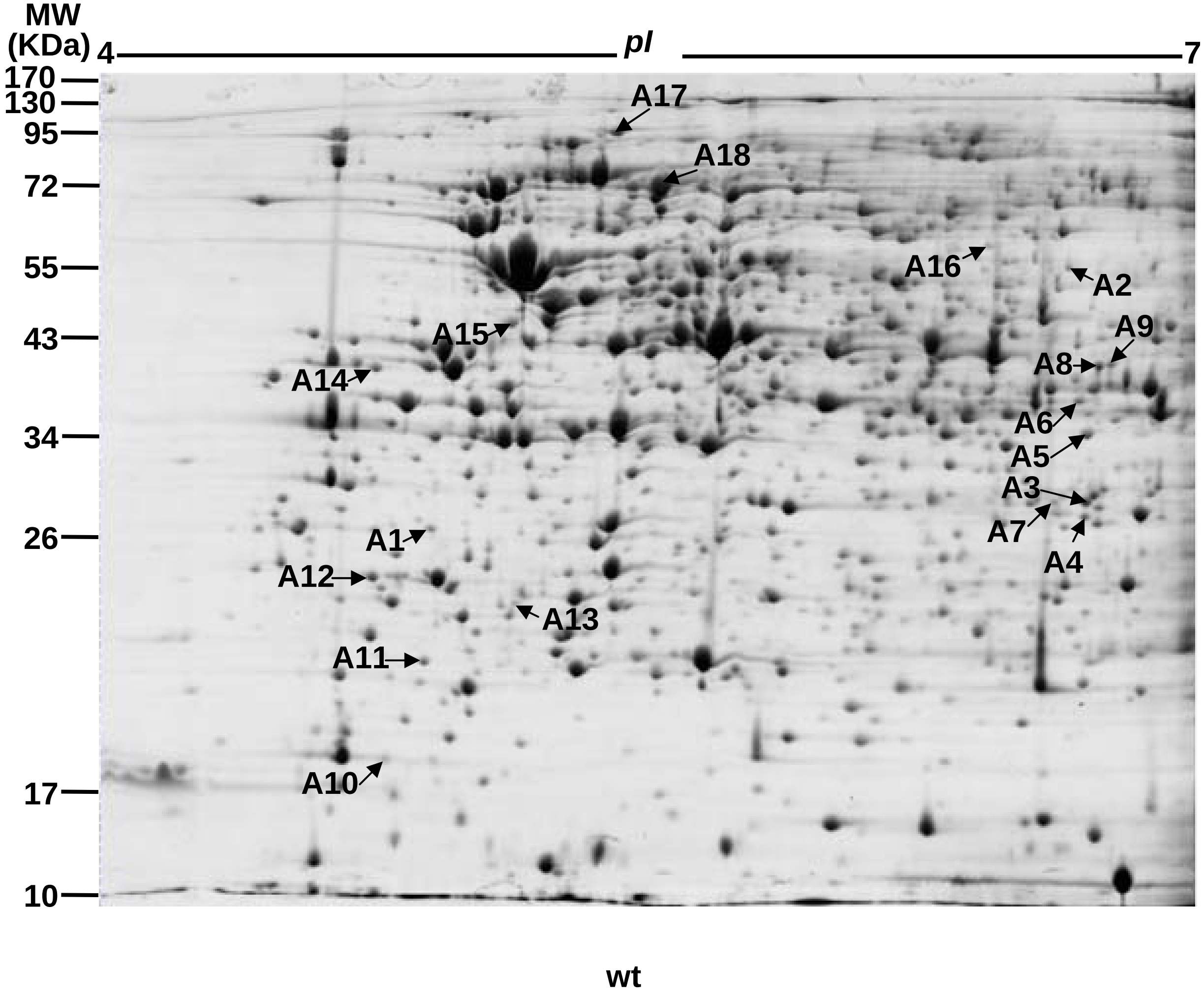

Additional file 8B

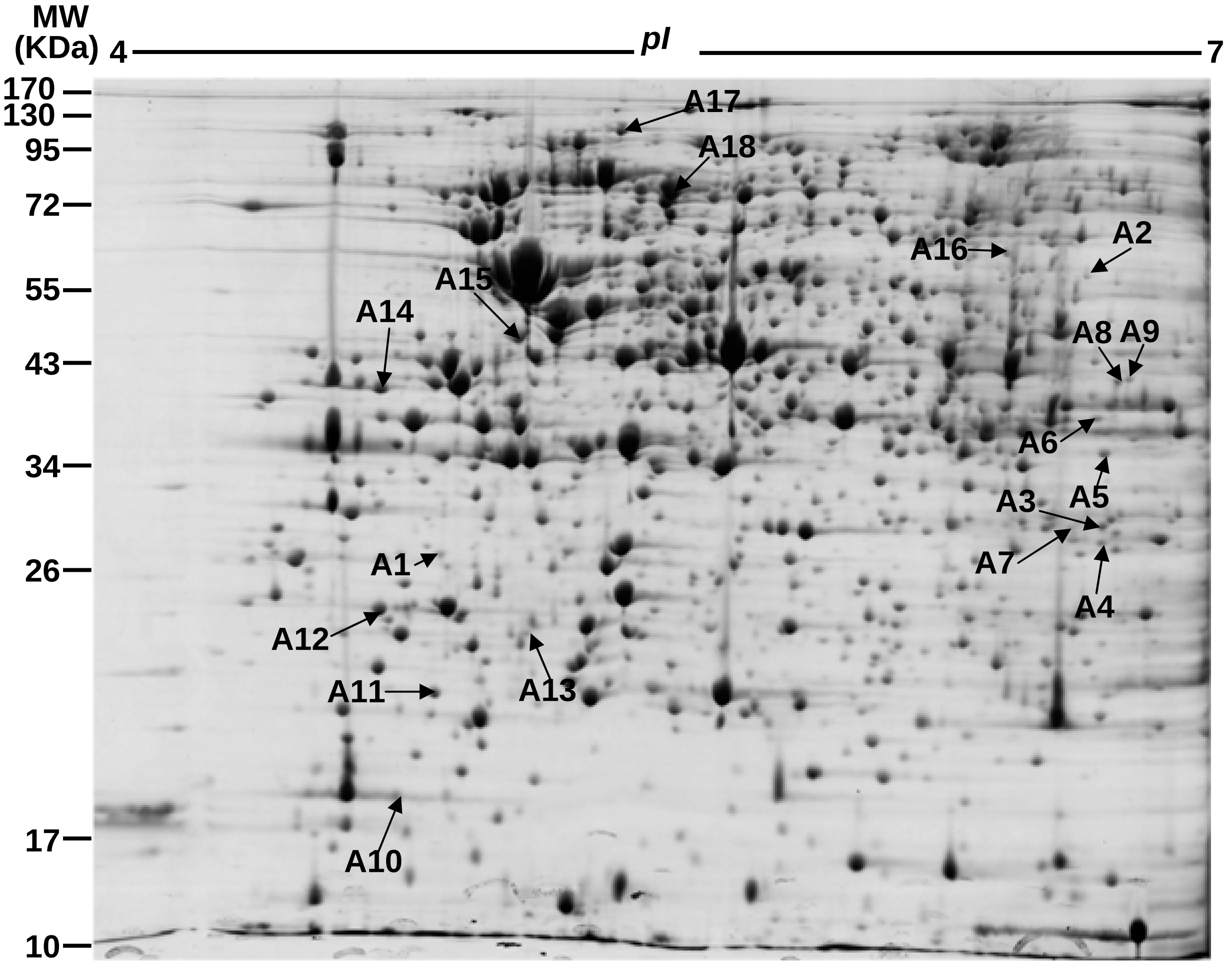

△sRNA-*Xoo*4 mutant
