# Supplementary material for: Identification and functional characterization of small non-coding RNAs in Xanthomonas oryzae pathovar oryzae
Source: BMC Genomics. 2011 Jan 30;12:87. doi: 10.1186/1471-2164-12-87 (PMC3039613; doi:10.1186/1471-2164-12-87)
Supplement: Additional file 9 — Differentially expressed proteins in ΔsRNA-Xoo3 and ΔsRNA-Xoo4 identified by MS (pdf). [file 1471-2164-12-87-S9.PDF]

Additional file 9: Differentially expressed proteins in △sRNA-*Xoo*3 and △sRNA-*Xoo*4 identified by MS

spot ID	protein name	function	NCBI acc.no.	mascot Score	sequence coverage	theoretical MW(Da)/pI	ratio± SD
**Down-regulated protein spots in sRNA-*Xoo*3mutant**							
C1 4720	type II citrate synthase	metabolism	PXO_02359	701	64%	49335/5.8	6.59 ± 1.31
C2 5273	tryptophan repressor binding protein	transcription regulation	PXO_03044	438	52%	20167/6.05	5.33 ± 0.62**
C3 4850	glyceraldehyde-3-phosphate dehydrogenase, type I	oxidation reduction	PXO_02308	529	69%	36134/6.35	5.31 ± 0.95
C4 5042	2-hydroxyhepta-2,4-diene-1, 7-dioateisomerase/5-carboxymethyl-2-oxo-hex-3-ene-1, 7-dioatedecarboxyl	metabolism	PXO_03265	656	73%	30784/5.2	5.04 ± 0.24**
C5 5065	septum site-determining protein MinD	DNA replication	PXO_04464	690	69%	29008/5.21	3.52 ± 0.44**
C6 5240	superoxide dismutase	oxidation reduction	PXO_00389	343	68%	22703/5.47	3.41 ± 0.54**
C7 4690	argininosuccinate synthase	amino-acid biosynthesis	PXO_00352	769	67%	45217/5.66	3.36 ± 0.46**
C8 5106	50S ribosomal protein L25/general stress protein Ctc	translation	PXO_04541	425	59%	19946/4.71	2.42 ± 0.49
C9 4802	DNA-directed RNA polymerase subunit alpha	transcription	PXO_04496	519	59%	36455/5.58	2 ± 0.17**
C10 5362	low molecular weight heat shock protein	response to stress	PXO_02640	302	74%	17910/6.07	1.55 ± 0.16**
C14 3382	ferrochelatase	heme biosynthetic process	PXO_03842	358	57%	35879/6.11	1.55 ± 0.2**
C15 3417	phenylalanine 4-monooxygenase	oxidation reduction	PXO_03285	326	34%	33469/5.12	1.53 ± 0.2**
C16 3800	low molecular weight heat shock protein	response to stress	PXO_02640	306	79%	17910/6.07	1.5 ± 0.07**
C17 3741	peptide deformylase	protein biosynthesis	PXO_02433	228	28%	19123/4.87	1.48 ± 0.09**
C19 2943	Malate dehydrogenase	tricarboxylic acid cycle	PXO_02545	541	62%	34946/5.37	3.51 ± 1.08
C22 4819	alcohol dehydrogenase	NADH oxidation	PXO_03296	619	65%	38180/6.05	4.97 ± 1.09
C23 4622	Putative reductase	oxidation reduction	PXO_03458	638	64%	44269/5.33	3.94 ± 0.59**
C24 4404	dihydrolipoamide dehydrogenase	tricarboxylic acid cycle	PXO_03103	597	56%	63323/5.78	2.97 ± 0.29**
**Up-regulated protein spots in sRNA-*Xoo*3 mutant**							
C25 4552	ATP synthase subunit alpha	ATP synthesis	PXO_03111	640	48%	55391/5.38	2.97 ± 0.7
C26 4500	mannose-1-phosphate guanylyltransferase/mannose-	lipopolysaccharide biosynthetic	PXO_03173	413	53%	51100/5.81	1.95 ± 0.56

	6-phosphate isomerase	process					
C11 4282	periplasmic beta-glucosidase	carbohydrate metabolic process	PXO_04104	817	56%	77571/4.95	1.45 ± 0.04**
C12 4513	mannose-1-phosphate guanylyltransferase/mannose-6-phosphate isomerase	lipopolysaccharide biosynthetic process	PXO_03173	930	74%	51100/5.81	1.43 ± 0.02**
C13 4798	acyl-CoA dehydrogenase family member 8	Fatty acid metabolism	PXO_04805	473	65%	42544/6.03	1.41 ± 0.01**
C18 3166	hypothetical protein	hypothetical protein	PXO_03595	701	81%	35275/5.53	1.61 ± 0.12**
C20 2979	glutathione synthetase	glutathione metabolism	PXO_01601	550	73%	34601/5.56	1.91 ± 0.46
C21 2511	alkaline phosphatase	metabolic pathways	PXO_00684	504	58%	67370/5.98	1.64 ± 0.37
**Down-regulated protein spots in sRNA-*Xoo*4 mutant**							
A1 5046	septum site-determining protein MinD	DNA replication	PXO_04464	474	62%	29008/5.21	1.443 ± 0.02**
A2 4489	alpha-L-fucosidase	metabolic process	PXO_01643	563	56%	59259/6.18	1.829 ± 0.16**
A3 5006	lipoprotein, putative	cell outer member	PXO_04714	661	62%	36108/7.64	3.009 ± 0.19**
A4 5027	ribosomal small subunit pseudouridylate synthase	rRNA processing	PXO_03083	690	80%	25844/6.24	1.76 ± 0.30**
A5 4899	Uroporphyrinogen decarboxylase	metabolic process	PXO_02179	571	71%	38536/6.16	1.97 ± 0.13**
A6 4818	Chorismate synthase	biosynthesis	PXO_01260	523	56%	39356/6.16	2.1 ± 0.21**
A7 5008	lipoprotein	cell outer member	PXO_04714	428	62%	36108/7.64	2.22 ± 0.22**
A8 4737	chaperone protein DnaJ	DNA replication	PXO_01186	692	60%	41079/6.21	2.93 ± 0.12**
A9 4733	4-hydroxy-3-methylbut-2-en-1-yl diphosphate synthase	biosynthesis	PXO_00800	459	67%	41028/5.79	2.04 ± 0.15**
**Up-regulated protein spots in sRNA-*Xoo*4 mutant**							
A10 5319	conserved hypothetical protein	hypothetical protein	PXO_03968	331	49%	21463/4.82	1.466 ± 0.08**
A11 5243	hypothetical protein	hypothetical protein	PXO_00915	568	53%	21779/4.87	1.478 ± 0.10**
A12 5112	hypothetical protein	hypothetical protein	PXO_02098	569	41%	39784/9.12	1.416 ± 0.086
A13 5156	two-component system regulatory protein	two-component signal transduction	PXO_03258	351	55%	24094/5.01	1.58 ± 0.14**
A14 4739	fimbrial assembly membrane protein	Protein secretion	PXO_02354	481	43%	34185/4.52	1.93 ± 0.15**
A15 4631	outer membrane protein	transport	PXO_03551	649	58%	47410/5.27	1.55 ± 0.085**
A16 4435	electron transfer flavoprotein-ubiquinone oxidoreductase	transport	PXO_03178	455	64%	59937/5.94	1.55 ± 0.12**
A17 2570	TonB-dependent receptor	transport	PXO_02600	839	60%	106642/5.68	2.21 ± 0.12**
A18 2728	Polyribonucleotide nucleotidyltransferase	RNA processing	PXO_01307	719	55%	75502/5.47	2.3 ± 0.22**

SD: Standard Deviation. Statistical significance was determined using Student's two-tailed *t* test for unpaired means $p<0.05$, $P<0.01$**.
