# Supplementary material for: Identification and functional characterization of small non-coding RNAs in Xanthomonas oryzae pathovar oryzae
Source: BMC Genomics. 2011 Jan 30;12:87. doi: 10.1186/1471-2164-12-87 (PMC3039613; doi:10.1186/1471-2164-12-87)
Supplement: Additional file 11 — Strains and plasmids used in this study (pdf). [file 1471-2164-12-87-S11.PDF]

Additional file 11: Strains and plasmids used in this study

**name**	**characteristics**	**source**
Xanthomonas oryzae* pv. *oryzae		
PXO99	wild type	our lab
△*hfq*	*hfq* inframe deletion strain	This study
△sRNA-*Xoo*1	sRNA-*Xoo*1 deletion strains	This study
△sRNA-*Xoo*2	sRNA-*Xoo*2 deletion strains	This study
△sRNA-*Xoo*3	sRNA-*Xoo*3 deletion strains	This study
△sRNA-*Xoo*4	sRNA-*Xoo*4 deletion strains	This study
△sRNA-*Xoo*5	sRNA-*Xoo*5 deletion strains	This study
△sRNA-*Xoo*6	sRNA-*Xoo*6 deletion strains	This study
△sRNA-*Xoo*7	sRNA-*Xoo*7 deletion strains	This study
Escherichia coli		
DH10B	F- *mcr*A Δ(*mrr*-*hsd*RMS-*mcr*BC) φ80*lac*ZΔM15 Δ*lac*X74 *rec*A1 *end*A1 *ara*D139 Δ(*ara*, *leu*)7697 *gal*U *gal*K λ- *rps*L *nup*G	Invitrogen
**Plasmids**		
pGEM-T	T-vector	Promega
pSPROT1	vector	Invitrogen
pk18mobsacB	empty vector	Our lab
pkms-hfq	deletion of the *hfq* gene	This study
pkms02602-02603	deletion of the sRNA-*Xoo*1	This study
pkms01686-01687	deletion of the sRNA-*Xoo*2	This study
pkms03613-03614	deletion of the sRNA-*Xoo*3	This study
pkms02847-05774	deletion of the sRNA-*Xoo*4	This study
pkms00353-00354	deletion of the sRNA-*Xoo*5	This study
pkms-sRNA6ABCD	deletion of the sRNA-*Xoo*6	This study
pkms04745-04746	deletion of the sRNA-*Xoo*7	This study
pkms04361-04362	deletion of the sRNA-*Xoo*8	This study
