# Supplementary material for: Identification and functional characterization of small non-coding RNAs in Xanthomonas oryzae pathovar oryzae
Source: BMC Genomics. 2011 Jan 30;12:87. doi: 10.1186/1471-2164-12-87 (PMC3039613; doi:10.1186/1471-2164-12-87)
Supplement: Additional file 12 — Oligonucleotides used in this study (pdf). [file 1471-2164-12-87-S12.PDF]

Additional file 12: Oligonucleotides used in this study

name	sequence 5′ to 3′	used for	experiment
oligoG	TCGCGAGCGGCCGCGGGGGGGGGGGGGG	reverse transcription	library construction
Probe1	GCAGGGGCGGTAGGATTCGAACC	hybridization	library screen
Probe2	GGTGCCCAGAAGAGGACTCGAACCT	hybridization	library screen
Probe3	GTGCCCAAGAGGGGACTCGAACC	hybridization	library screen
Probe4	TGGCGGAGAGAGGGGGATTCGAAC	hybridization	library screen
Probe5	TGGTAGCGGGGGCAGGATTTGAAC	hybridization	library screen
Probe6	GCTCGAACCCCTGTTTTAGCCTT	hybridization	library screen
Probe7	AACCTGTAACCGCCGGCTTAGAA	hybridization	library screen
Probe8	GGATTCGAACCACCGAAGGCGTAA	hybridization	library screen
Probe9	GATGACCTACTCTCGCATGGCTTG	hybridization	library screen
Probe10	GGTCAAACCGCACGGATCATTAGTATCAG	hybridization	library screen
Probe11	GAACGTCAAGCACGACAGGGTGG	hybridization	library screen
Probe12	CGACCAGTGAGCTATTACGC TTTCT	hybridization	library screen
Probe13	GTCTACTGCCCGCGACTATGC	hybridization	library screen
Probe14	TGATCCAGCCGCACCTTCCG ATAC	hybridization	library screen
Probe15	TGCACCCAACATCCAGTTCGCATCG	hybridization	library screen
00157-00156up (F1)	GTTGAAGGTATGGAACGCGATG	*hfq*	CT analysis
00157-00156down (R1)	GCAACTGGATCTACGCGAGCTA	*hfq*	CT analysis
00156-00155up (F2)	CCATCAATTGGAGCGCGCACTT	*hfq*	CT analysis
00156-00155down (R2)	CATTGACCAGATACACCGACAC	*hfq*	CT analysis
00155-00154up (F3)	CGGGTGGTGGGTATGTGCAATC	*hfq*	CT analysis
00155-00154down (R3)	CGACGGGCTTGGTTTGTCGATA	*hfq*	CT analysis
00154-00153up (F4)	GATCGCCGCGAAGACTGGGAAG	*hfq*	CT analysis
00154-00153down (R4)	CAGCTTTCGGCAGTGACCAGGC	*hfq*	CT analysis
2945upper	ATC**GAATTC**CAGCAGTTGCGGTAGATCG	*hfq*	*hfq* deletion
2945down	ATT**GCATGC**CGGAGTAGTGCGTGTTTGACC	*hfq*	*hfq* deletion
2947upper	ATC**GCATGC**CCCCAGCCGGTCCCGGAAG	*hfq*	*hfq* deletion
2947down	ATT**AAGCTT**GCGCTGGCGCTGGAAGCAG	*hfq*	*hfq* deletion
pHM1-hfq-up	TAT**GGTACC**ATGCCAGGTGTCGACCGGGAC	*hfq*	complementary
pHM1-hfq-down	GAT**GAGCTC**TTACTGCTCGACGTCGTCA	*hfq*	complementary
0947up	ATT**GAATTC**ACTCCGAAAGAGCATCTGG	sRNA-*Xoo*1	sRNA deletion

0947down	ATT**CTGCAG**TCTGCGATTCAGCTAAACC	sRNA-*Xoo*1	sRNAs deletin
0948up	ATT**CTGCAG**GCACCAATTGGATGCATGA	sRNA-*Xoo*1	sRNA deletion
0948down	ACC**AAGCTT**ATACGGATGCAGGTACAGC	sRNA-*Xoo*1	sRNA deletion
1823up1	ATC**GAATTC**CTGATCCTTGCAAGGCACGC	sRNA-*Xoo*2	sRNA deletion
1823down1	ATT**GGATCC**TGTGCCCGCATGCCGAATGA	sRNA-*Xoo*2	sRNA deletion
1824up2	ATT**GGATCC**GTTACTTCGCCTTCAACACG	sRNA-*Xoo*2	sRNA deletion
1824down2	ATT**AAGCTT**TTGTTTCTCCGATGCTGGTC	sRNA-*Xoo*2	sRNA deletion
0247up	ACT**GAATTC**TTAAATTCTTCGACATTCC	sRNA-*Xoo*3	sRNA deletion
0247down	TCA**GGATCC**TCATTGATTTTGTTTAGCA	sRNA-*Xoo*3	sRNA deletion
0248up	ATT**GGATCC**ATGGCAACGGTGGGTCGCT	sRNA-*Xoo*3	sRNA deletion
0248down	ATT**AAGCTT**GGAGCTGTTTCCGCGCGAG	sRNA-*Xoo*3	sRNA deletion
0431up	ATT**GAATTC**CTACTTTATCTGATCGTGA	sRNA-*Xoo*4	sRNA deletion
0431down	ATT**GGATCC**CTCACTCAGTATACAGCGT	sRNA-*Xoo*4	sRNA deletion
0432up	ATT**GGATCC**CACCGCGTCGACTGCATAT	sRNA-*Xoo*4	sRNA deletion
0432down	ATT**CTGCAG**TGTGCGACGGCGCGTTGGG	sRNA-*Xoo*4	sRNA deletion
2676up1	ATT**GAATTC**TGCCCGGCTGCAGTACCACC	sRNA-*Xoo*5	sRNA deletion
2676down1	ATT**GGATCC**GACGTTGAAGACCCAGCCTA	sRNA-*Xoo*5	sRNA deletion
2677up2	ATT**GGATCC**TCGCACGCATGTGGGCACGG	sRNA-*Xoo*5	sRNA deletion
2677down2	ATT**AAGCTT**GATGGTGATCGAGAACGTGA	sRNA-*Xoo*5	sRNA deletion
D-sRNA6-A	ATT **GAATTC** ATCGGGGCGTTGGCATTCTTG	sRNA-*Xoo*6	sRNA deletion
D-sRNA6-B	ATT **GGATCC** TGGTGGCTAGGGTCAAGTTGC	sRNA-*Xoo*6	sRNA deletion
D-sRNA6-C	ATT **GGATCC** CCAGCCCATGGCAAGGAGAG	sRNA-*Xoo*6	sRNA deletion
D-sRNA6-D	ATT **AAGCTT** TGCCCAAATCCCCCAGCACAT	sRNA-*Xoo*6	sRNA deletion
1471up1	ATT **GAATTC** AACAATTGCAGGCCGGTGG	sRNA-*Xoo*7	sRNA deletion
1471down1	ATT **GTCGAC** TTCGTTTTCTGCGCCTGCTT	sRNA-*Xoo*7	sRNA deletion
1472up2	ATT **GTCGAC** GCGACAGTTACCTGTGTGCT	sRNA-*Xoo*7	sRNA deletion
1472down2	ATT **AAGCTT** TGATTCCCGAGGATTATCTG	sRNA-*Xoo*7	sRNA deletion
3821up1	ATT GAATTC TTCCACGGTCTGCTTGATCC	sRNA-*Xoo*8	sRNA deletion
3821down1	ATT GGATCC ACAGCGTGACCGGGTGATGA	sRNA-*Xoo*8	sRNA deletion
3822up2	ATT GGATCC GACGCGGCCGACGCTCCTTA	sRNA-*Xoo*8	sRNA deletion
3822down2	ATT GTCGAC ACATCACCGCCGGCCCGGAC	sRNA-*Xoo*8	sRNA deletion
IGR784 probe	GGCGCGAAGGATACGTCGTAATTATTTGAATTCGTCGGCG	sRNA-*Xoo*1	Northern blot
IGR1487 probe1	GGACCCGACGAGGTAGTCCGCCGCTCAGTGGCTGC	sRNA-*Xoo*2	Northern blot
0247-0248 probe	CCCGGTGGACAATGAGTGGCACAGCTTCCGACTGAC	sRNA-*Xoo*3	Northern blot
IGR309 probe	GCGTCCGTCTTCCTTGAAGCCAGAAACAAGACTCG	sRNA-*Xoo*4	Northern blot
2676-2677 probe	CCGGGCGGCGCTTGTGTCGTGCTGAAAGGCGTTTGC	sRNA-*Xoo*5	Northern blot
4616-4617 probe	CGAGAACGCCGAGCCGTGCCAAGCCTTTTG	sRNA-*Xoo*6	Northern blot
1471-1472 probe	TCAAGGCCTGAGGTCGTGCTCGGTGTCGTCATCGAACGCG	sRNA-*Xoo*7	Northern blot

3821-3822 probe	GCTGCCGGATCGGCACCGATCCGGCAGAAATGACA	sRNA-*Xoo*8	Northern blot
0047-0048 probe	CGCGCGGTGTCTGTCGGTGAGCGAATGTGGTCCAGC	PXO 03433-03534	Northern blot
2400-2401 probe	ACGCCTCAGGACGTCAGGTGTTTGCGTGCGTTGCCG	PXO 00729-00730	Northern blot
3072-3073 probe	AATCATCGGAAGTCAGGCTTCTGGCCAATCAACTTC	PXO 01517-01518	Northern blot
IGR2202 probe	GCCAGGCCGTCACGCGCAATATACTTTGTTCAACT	PXO 00416-00417	Northern blot
IGR2157-probe	TCGCACTTACCCTCACGCCACTCCCTCTCCCGGTG	PXO 00355-00356	Northern blot
5' RACE Inner	CGCGGATCCGAACACTGCGTTTGCTGGCTTTGATG	universal primer	5′ RACE
5'RACE Outer	GCTGATGGCGATGAATGAACACTG	universal primer	5′ RACE
784-down -outer	CCAGAAGGCGCGAAGGATAC	sRNA-*Xoo*1	5′ RACE
1487-down-outer	GACGAGGTAGTCCGCCGCTCA	sRNA-*Xoo*2	5′ RACE
RACE-IGR1487	AAGCGGCGGACCCGACGAGGTA	sRNA-*Xoo*2	5′ RACE
RACE-177	GCACAAGGCGCGATCCCTTCGA	sRNA-*Xoo*3	5′ RACE
18A4-down-outer	TCCGTCTTCCTTGAAGCCAGAAAC	sRNA-*Xoo*4	5′ RACE
18A4-down-inner	AAGACTCGGTCGTAGTACAAATCT	sRNA-*Xoo*4	5′ RACE
RACE-IGR1930	TACCCAGCGAAAATGTGGGATT	sRNA-*Xoo*5	5′ RACE
NC70down-outer	GCT TAT TCC CCT TGC GGG TGT T	sRNA-*Xoo*6	5′ RACE
NC19D-OUTER	GGACAGTCCACAGGACAAATGA	sRNA-*Xoo*7	5′ RACE
NC19D-INNER	ATCAGTGCTTGAGCAGCGATTC	sRNA-*Xoo*7	5′ RACE
